# An adoptive cell therapy with TREM2‐overexpressing macrophages mitigates the transition from acute kidney injury to chronic kidney disease

**DOI:** 10.1002/ctm2.70252

**Published:** 2025-02-25

**Authors:** Yating Zhang, Yu Liu, Siweier Luo, Hanzhi Liang, Chipeng Guo, Yufei Du, Hongyu Li, Le Wang, Xiaohua Wang, Chun Tang, Yiming Zhou

**Affiliations:** ^1^ Basic and Translational Medical Research Center, Sun Yat‐sen Memorial Hospital Sun Yat‐sen University Guangzhou Guangdong China; ^2^ Guangdong Provincial Key Laboratory of Malignant Tumor Epigenetics and Gene Regulation, Guangdong‐Hong Kong Joint Laboratory for RNA Medicine, Sun Yat‐sen Memorial Hospital Sun Yat‐sen University Guangzhou Guangdong China; ^3^ Department of Nephrology, The Seventh Affiliated Hospital of Sun Yat‐sen University Sun Yat‐sen University Shenzhen Guangdong China; ^4^ Department of Nephrology, The First Affiliated Hospital Sun Yat‐sen University Guangzhou Guangdong China

**Keywords:** AKI–CKD transition, cell therapy, kidney disease, macrophages, TREM2

## Abstract

**Background:**

Macrophages have been shown to contribute to renal injury and fibrosis as well as repair. Recently, Triggering Receptor Expressed on Myeloid Cells 2 (TREM2)‐positive macrophages have been shown to play important roles in regulating tissue inflammation and repair. However, it remains unclear whether they can mitigate the transition from acute kidney injury to chronic kidney disease (the AKI–CKD transition).

**Methods:**

The AKI–CKD transition was generated by unilateral ischaemia–reperfusion injury (UIRI) in wild‐type (WT) and Trem2 knockout mice. F4/80 magnetic beads were used to isolate renal macrophages. Flow cytometry was used to determine the levels of TREM2 and CD11b levels. Quantitative reverse transcription polymerase chain reaction (qRT‐PCR), Western blotting and histological staining were performed to determine the expression of cytokines and fibrotic markers. RNA‐seq was used to investigate transcriptomic changes between WT and Trem2 knockout bone marrow‐derived macrophages (BMDMs). TREM2‐overexpressing macrophages were generated using lentivirus and transferred intravenously to UIRI mice.

**Results:**

TREM2 macrophages exhibited a strong renal protective effect on the AKI–CKD transition. Genetic deletion of Trem2 resulted in increased renal inflammation and exacerbated renal injury and fibrosis in UIRI mice. Interestingly, we found that hypoxia could increase TREM2 expression in macrophages via HIF‐1α. Upregulated TREM2 expression enhanced macrophage phagocytosis and suppressed the expression of pro‐inflammatory cytokines, resulting in lower levels of apoptosis and fibrosis in tubular epithelial cells. Using RNA‐seq analysis, we showed that the regulatory effects of TREM2 were orchestrated by the PI3K‐AKT pathway. Pharmacological regulation of the PI3K‐AKT pathway could modulate the macrophage‐mediated inflammation and phagocytosis. In addition, an adoptive cell therapy using TREM2‐overexpressing macrophages effectively reduced the immune cell infiltration, renal injury and fibrosis in UIRI mice.

**Conclusion:**

Our study not only provides valuable mechanistic insights into the role of Trem2 in the AKI–CKD transition but also offers a new avenue for TREM2‐overexpressing macrophage‐based adoptive cell therapy to treat kidney diseases.

**Key points:**

- TREM2 knockout worsens kidney injury and accelerates AKI–CKD transition.
- TREM2 is upregulated by hypoxia via HIF1α in AKI–CKD transition.
- An adoptive cell therapy using TREM2‐overexpressing macrophages reduces kidney inflammation and fibrosis.

## Graphical abstract

In unilateral ischaemia–reperfusion injury (UIRI), Trem2 expression is upregulated by hypoxia via HIF‐1α in macrophages, and the activation of Trem2 enhances phagocytosis and reduces cytokine production through the PI3K‐AKT pathway in macrophages. An adoptive cell therapy using Triggering Receptor Expressed on Myeloid Cells 2 (TREM2)‐overexpressing macrophages effectively mitigates the transition from acute kidney injury (AKI) to chronic kidney disease (CKD) in UIRI animals.

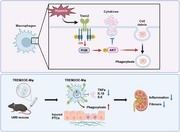

## 1 INTRODUCTION

Owing to their increasing impact on morbidity and mortality, acute kidney injury (AKI) and chronic kidney disease (CKD) are interrelated syndromes of contemporary concern.^1^, ^2^, ^3^ Studies indicate that AKI could increase the risk of CKD development, with the likelihood of progression depending on the severity of AKI. A meta‐analysis study has shown that, compared with individuals without AKI, patients with AKI have approximately nine‐, three‐ and two‐times higher risk of developing CKD, ESKD and mortality, respectively.^4^ AKI is defined by a sudden and temporary decline in renal function, marked by a rapid reduction in the glomerular filtration rate (GFR) and increased levels of serum creatinine (CREA), blood urea nitrogen (BUN) and electrolytes. If this condition continues for more than 3 months, it can lead to the development of CKD or renal failure.^5^, ^6^ A recent study of more than 2 million patients with AKI confirmed that these patients are more susceptible to CKD development.^7^, ^8^ The transition from AKI to CKD may involve various factors, such as hypoxia, chronic inflammation, tubular epithelial injury and fibrosis.^9^, ^10^, ^11^

Immune cells, including macrophages and their products, have been shown to contribute to kidney injury and inflammation.^12^, ^13^, ^14^ Notably, in rodents with ischaemia–reperfusion injury (IRI), macrophages not only promote initial kidney injury but also promote fibrosis after IRI.^15^, ^16^, ^17^, ^18^ These observations were further supported by evidence that depletion of monocytes/macrophages with liposomal clodronate before ischaemic injury attenuated kidney fibrosis.^19^, ^20^, ^21^, ^22^ While macrophages promote inflammation in the early stages of renal injury, there is also evidence that they play an important role in tissue repair by scavenging cellular debris and regulating regeneration.^23^

Triggering Receptor Expressed on Myeloid Cells 2 (TREM2) is a membrane protein that was originally found in mouse dendritic cells and macrophages.^24^ TREM2 belongs to the immunoglobulin superfamily of single transmembrane receptors and binds to lipid molecules, including phospholipids, lipopolysaccharide (LPS) and apolipoproteins. Intracellularly, TREM2 binds to the adaptor proteins DAP10 and DAP12, which can activate the Spleen Tyrosine Kinase (SYK).^25^, ^26^ This signalling triggers a cascade of protein phosphorylation leading to Ca^2+^ mobilisation, integrin activation and cytoskeletal rearrangement, which regulate macrophage phagocytosis and the release of inflammatory factors.^27^ TREM2 is highly expressed in the monocyte/macrophage system in different organs, including the central nervous system, liver, kidney and tumours.^28^, ^29^, ^30^ Studies have suggested that TREM2 may be involved in regulating inflammatory responses and cellular stress responses in kidney disease. Specifically, TREM2 may influence the development of kidney injury by regulating macrophage phagocytosis and the release of inflammatory factors.^31^, ^32^, ^33^, ^34^ After interaction with DAP12, activation of TREM2 stimulates the downstream PI3K‐AKT pathway,^35^, ^36^, ^37^ leading to an increase in levels of intracellular calcium ions,^38^ which further affects macrophage phagocytic function and the inflammatory response.^39^

Recent studies suggest that TREM2 macrophages play crucial roles in tissue inflammation and repair. However, whether they contribute to the AKI‐to‐CKD transition remains unknown. In this study, we identified a renal protective role of TREM2+ macrophages in the AKI–CKD transition. Our findings show that the number of renal TREM2+ macrophages increases in a unilateral ischaemia‐reperfusion injury (UIRI)‐induced AKI–CKD transition mouse model. Genetic deletion of Trem2 in mice resulted in increased levels of tissue inflammation and fibrosis and exacerbated kidney injury after UIRI. Mechanistically, we found that hypoxia could regulate TREM2 expression in macrophages via HIF‐1α. Activation of TREM2 promoted macrophage phagocytosis while reducing pro‐inflammatory cytokine expression simultaneously. RNA‐seq analysis identified the PI3K‐AKT signalling pathway as a key mediator of these effects. Moreover, inhibition of p‐AKT activity with pharmacological treatment eliminated TREM2‐induced changes in macrophages. Notably, an adoptive cell therapy with mouse macrophages overexpressing TREM2 effectively reduced renal injury and fibrosis in UIRI mice. In conclusion, our study not only provides valuable mechanistic insights into the role of Trem2 in regulating macrophage‐mediated inflammation and fibrosis in the AKI–CKD transition but also offers a new avenue for TREM2‐overexpressing macrophage‐based cell therapy to treat kidney diseases.

## 2 METHODS

### 2.1 Animals

All the animals were kept in a standard specific‐pathogen free (SPF) facility at Sun Yat‐sen Memorial Hospital, Sun Yat‐sen University. The Trem2 knockout (Trem2KO) mice were a gift from Professor Yingping Guo at Southern Medical University.^40^ Male C57BL/6 Trem2KO and wild‐type (WT) littermate mice aged 6–10 weeks were used in this study. For the UIRI model, the left renal pedicles of the mice were clipped for 45 min. Mice body temperature was maintained at 37°C during ischaemia. Animals were sacrificed after metabolic collection on day 14. Blood samples and kidney tissues were collected. All procedures were in accordance with the NIH Guidebook for the Care and Use of Laboratory Animals and approved by the Ethics Committee of Sun Yat‐sen Memorial Hospital (No. AP20230083).

### 2.2 H&E staining

The paraffin sections were deparaffinised and stained with Harris haematoxylin for 10 s, followed by thorough rinsing with running tap water until the excess stain was removed. The sections were then counterstained with Eosin for 30 s and rinsed until the excess stain was removed. Sections were then treated with a series of alcohol solutions (50%, 70%, 80%, 95% twice and 100% twice) and with Xylene three to four times. Finally, the sections were mounted on glass slides using Permount and covered with a coverslip. The haematoxylin and eosin (H&E)‐stained images were taken using a Nikon light microscope and were analysed using ImageJ software.

### 2.3 Periodic‐Acid Schiff (PAS) staining

The paraffin sections were sequentially deparaffinised in environmentally friendly deparaffinising solution, placed in 75% alcohol for 5 min and then in PAS staining solution B for staining, subjected to PAS staining solution A for immersion staining, protected from light, and rinsed with tap water. The sections were placed in PAS staining solution C for staining, differentiated using aqueous hydrochloric acid solution, subjected to ammonia for bluing, and finally rinsed under running water. Dehydration was performed, followed by transparency, and finally, the slices were sealed with neutral gum.

### 2.4 Masson staining

The sections were deparaffinised sequentially in xylene I and xylene II followed by alcohol and then washed with tap water. These samples were then incubated with Masson liquid (DC0032, Leagene) overnight, rinsed, treated with 1% glacial acetic acid and then placed into two vats of anhydrous ethanol for dehydration. The sections were treated with xylene solution for transparency.

### 2.5 Sirius Red staining

Deparaffinised tissue sections were stained with azurite blue solution and Sirius Red saturated picric acid solution (PH1099, Phygene) for 15–30 min. Dehydration and differentiation were performed, and the sections were treated with xylene solution for transparency.

### 2.6 Immunohistochemistry

Kidney sections were blocked using the goat serum (ZLI‐9021, SolelyBio), followed by incubation with the primary antibody for 12 h at 4°C. The sections were washed with PBS‐Tween‐20 three times and incubated with horseradish peroxidase (HRP) secondary antibodies and incubated at room temperature for 2 h. The staining was visualised using 3,3′‐Diaminobenzidine (DAB) solution (ZLI‐9017, SolelyBio). The positive signals in the periglomerular and tubulointerstitial regions were achieved by manually selecting regions of interest (ROIs) based on the anatomical landmarks visible in the tissue sections. In total, 20 fields at 20× magnification view were randomly selected for each kidney, and their mean values were calculated with ImageJ. Specifically, periglomerular regions were defined around the glomeruli, while the tubulointerstitial areas were delineated based on the proximity to tubules and interstitial spaces. We then used ImageJ to quantify the positive staining areas within these designated ROIs.

### 2.7 Isolation of macrophages with F4/80 magnetic beads

Whole kidneys were freshly removed and cut into small pieces. Single‐cell dissociation was performed with 0.25 mg/mL Liberase enzyme (5401119001, Roche) for 45 min at 37°C. After enzymatic digestion, single cells were filtered through a 40 µm cell strainer and washed with cold PBS. Red blood cells (RBCs) were removed with precooled RBC lysate at 4°C. The macrophages were then isolated with F4/80 magnetic beads (100‐0659, Stemcell Technologies) following the instructions supplied by the vendor. Single kidney cells were resuspended in isolation buffer at 1 × 10⁸ cells/mL and blocked with rat serum. They were then treated with selection solution for 5 min and incubated with the F4/80 beads (60 µL/mL) for 5 min at room temperature. After the mixture was kept on a magnetic rack for 5 min, the supernatant was discarded and the isolated macrophages were resuspended in PBS. This process was repeated three times to remove F4/80‐ cells. Finally, F4/80+ macrophages were counted and resuspended in complete Dulbecco's Modified Eagle Medium (DMEM).

### 2.8 Urinary albumin assay

The 24‐hour urine volume of each animal was measured. The 500 µL supernatant was transferred into new tube following centrifugation at 4000 rpm for 5 min. After adding 500 µL of methanol and 120 µL of chloroform into the supernatant, the mixture was centrifuged at 13 000 rpm for 10 min at 4°C. The pellet was washed with 600 µL of methanol and centrifuged at 15 000 rpm for 10 min at 4°C. The protein pellet was allowed to air‐dry and was resuspended in 50 µL of 1× SDS loading buffer. After denatured for 5 min at 100°C, proteins were separated by electrophoresis on a 4%–20% gradient gel with albumin standards. After staining with Coomassie Brilliant Blue for 5–10 min, the gel was washed overnight with deionised water. Urinary albumin concentration was determined using a standard curve from albumin standards.

### 2.9 Transmission electron microscopy

After the kidney tissues were collected, they were immediately placed in paraformaldehyde (PFA) fixative solution at 4°C for 5–10 min, immediately cut into 1 mm^3^ cross sections and placed in an ice box. Glutaraldehyde was used to fix the cell and tissue structure. The fixed kidney tissues were cut into extremely thin slices, usually between 70 and 90 nm thick, using an ultra‐thin microtome. The slices were and placed under a transmission electron microscope (TEM) for imaging.

### 2.10 In vitro hypoxia experiment

Immortalised bone marrow‐derived macrophages (iBMDMs; iCell Bioscience, icell‐0060a) were maintained in DMEM (C11995500BT, Gibco) with 10% fetal bovine serum (FBS; S‐FBS‐SA‐015, Serana Europe) and 1% antibiotics (15140122, Gibco). For hypoxia experiment, macrophages were cultured under hypoxic conditions (95% N_2_, 5% CO_2_) in a low‐glucose, FBS‐free medium at 37°C for 12 h. Following this, the cells were reoxygenated by transferring them to normoxic conditions (95% air, 5% CO_2_) in a high‐glucose medium supplemented with 10% FBS for an additional 12 h.

### 2.11 Lentiviral infection

The TREM2 gene (NM_031254.3) were cloned into a second‐generation lentiviral expression pSLenti‐vector. The lentivirus was produced by transfection of HEK293 cells for 48 h (OBIO). iBMDMs (5 × 10^4^ cells per well) were cultured in 24‐well plates for 12 h. GFP control and TREM2 lentiviruses were added to the cells at an MOI of 50 for 8–16 h. Cells were cultured for 3 days without changing medium. Infected cells were selected using culture medium supplied with 10 µg/mL blasticidin for 1 week before experiments.

### 2.12 Macrophage phagocytosis experiment

Macrophages were seeded in a 10 cm petri dish and incubated with 2.5 mg/mL pHrodo™ Red Zymosan (P35364, Thermo Fisher) for 30 min at 37°C. After washing with PBS three times, Zymosan fluorescent signals were analysed with the flow cytometry (BD FACSJazz) and the fluorescence microscope (Nikon Ts2R).

### 2.13 ROS assay

BMDMs were incubated with the ROS probe DCFH‐DA (5 µM, S0033S, Beyotime) at 37°C for 30 min. After three PBS washes, intracellular ROS fluorescence was analysed using flow cytometry (BD FACSJazz) and a fluorescence microscope (Nikon Ts2R). Relative fluorescence intensity was calculated using FlowJo and ImageJ softwares.

### 2.14 Real‐time qPCR

Total RNAs of tissues were extracted with TRIzol reagent (Invitrogen), and total RNAs of cells were isolated using an RNA rapid extraction kit (RN001, Yishan). cDNA was synthesised using RT Mix and quantified with SYBR Master Mix. The real‐time quantitative polymerase chain reaction (qPCR) was conducted using a CFX96 instrument (Bio‐Rad). The primers are shown in Table S1.

### 2.15 Western blotting

Proteins were isolated in the radioimmunoprecipitation assay (RIPA) buffer containing the cocktail inhibitors of protease and phosphatase. After bicinchoninic acid (BCA) quantification, protein samples were separated on 4%–12% gels and transferred to PVDF membranes. The membranes were then blocked with 5% bovine serum albumin (BSA) for 1 h, followed by overnight incubation at 4°C with primary antibodies and 1‐h incubation at room temperature with HRP‐conjugated secondary antibodies. Protein bands were detected using an enhanced chemiluminescence (ECL) kit and analysed using ImageJ software. The antibodies were shown in Table S2.

### 2.16 Flow cytometry

Flow cytometry experiments were performed using FACSVerse (BD Biosciences). Macrophage surface marker proteins including F4/80, CD11b and TREM2 were stained with appropriate antibodies (1:100 dilution) 20 min at room temperature. Stained macrophages were then washed with PBS three times before flow cytometry experiment. The flow cytometry results were analysed using a FlowJo v10.7.2 (TreeStar) software. The antibodies were shown in Table S2.

### 2.17 RNA‐seq

RNAs of BMDMs from WT and TREM2KO mice were collected using the Trizol reagent. After quality check, library was prepared and sequenced on an Illumina NovaSeq platform. After filtering out low‐quality reads, adapter sequences and poly‐N reads, the remaining clean reads were processed with fastp software to generate raw fastq data. Differentially expressed genes (DEGs) were revealed using the DeSeq2 method. Gene set enrichment analysis (GSEA) was conducted using Gene Ontology (GO) and Kyoto Encyclopedia of Genes and Genomes (KEGG) datasets with the analysis tool.

### 2.18 Macrophage adoptive transfer experiment

UIRI model was established with TREM2KO mice. On the second day of operation, 1 × 10^6^ GFP control‐iBMDM, TREM2OE‐iBMDM and equal volume of PBS were injected through the tail vein. The amount of iBMDM administrated was determined following previous reports.^41^, ^42^, ^43^ The 24‐h urine was collected using a metabolic cage on day 13, while serum and kidney samples were collected on day 14 for further experiments.

### 2.19 Statistics

Data are showed as means ± standard error of the mean (SEM), unless stated otherwise. For pairwise comparisons, the unpaired two‐tailed Student's *t*‐test was used. For multiple comparisons, one‐way analysis of variance (ANOVA) with Tukey's test was used. Statistical significance was set at *p* < .05, and all analyses were carried out using GraphPad Prism 8 software.

### 2.20 Ethics declarations

This study was approved by the Ethics Committee of Sun Yat‐sen Memorial Hospital (Approval number: AP20230083).

## 3 RESULTS

### 3.1 scRNA‐seq showing the upregulation of Trem2 expression in renal macrophages from AKI–CKD transition mice

To explore the changes in different macrophage populations during the transition from AKI to CKD, we specifically analysed the single‐cell transcriptomic changes in renal macrophages in IRI mice from a previously reported scRNA‐seq dataset.^44^ The count matrix after de‐batch analysis was downscaled using principal component analysis (PCA) according to the default 30 dimensions. Unified stream shape approximation and projection (UMAP) was then applied to cluster the cells. Cd11b+ macrophage subsets were then extracted. For the Cd11b+ macrophage subsets, we repeated the de‐batch analysis and clustering steps to eliminate technological inaccuracies and maintain the biological differences to distinguish the macrophages in the sham group from those in the fibrosis group at 7 days and 28 days after UIRI (Figure S1A–D). A total of 12 014 macrophages were used for the analysis. The UMAP of the macrophages was subsequently performed at a resolution of 0.1, and identification of the macrophage subsets was performed using the Wilcoxon test to obtain the identification markers from eight macrophage subsets (Figure S1E). All of the subsets expressed high levels of Cd14 and Cd11b, two well‐known markers of macrophages. Compared with those in the sham kidneys, the percentages of six macrophage clusters (clusters 1–6) in the kidneys were greater at 7 days and 28 days after UIRI (Figure S1F). DEG analysis revealed that 430 and 229 genes were upregulated and downregulated, respectively, in macrophages between fibrotic and sham kidneys (Figure S1G). Several genes, including *Trem2*, *Spp1*, *Apoe*, *Ptgs2*, *Vim* and *Il1b*, were significantly upregulated in macrophages from fibrotic kidneys (Figure S1H). The feature plots revealed that *Trem2* and *Spp1* were highly expressed in cluster 2; *Apoe* was expressed in clusters 0, 2 and 4; *Ptgs2* and *Il1b* were expressed in cluster 3; and *Vim* was highly expressed in clusters 1 and 2 (Figure S1I). Among these genes, *Trem2* was not expressed in macrophages from sham mice but was consistently upregulated in macrophages at 28 days after IRI, suggesting an important role for Trem2 in the AKI–CKD transition.

### 3.2 Trem2KO mice exhibit aggravated kidney injury and fibrosis after UIRI

**FIGURE 1:**
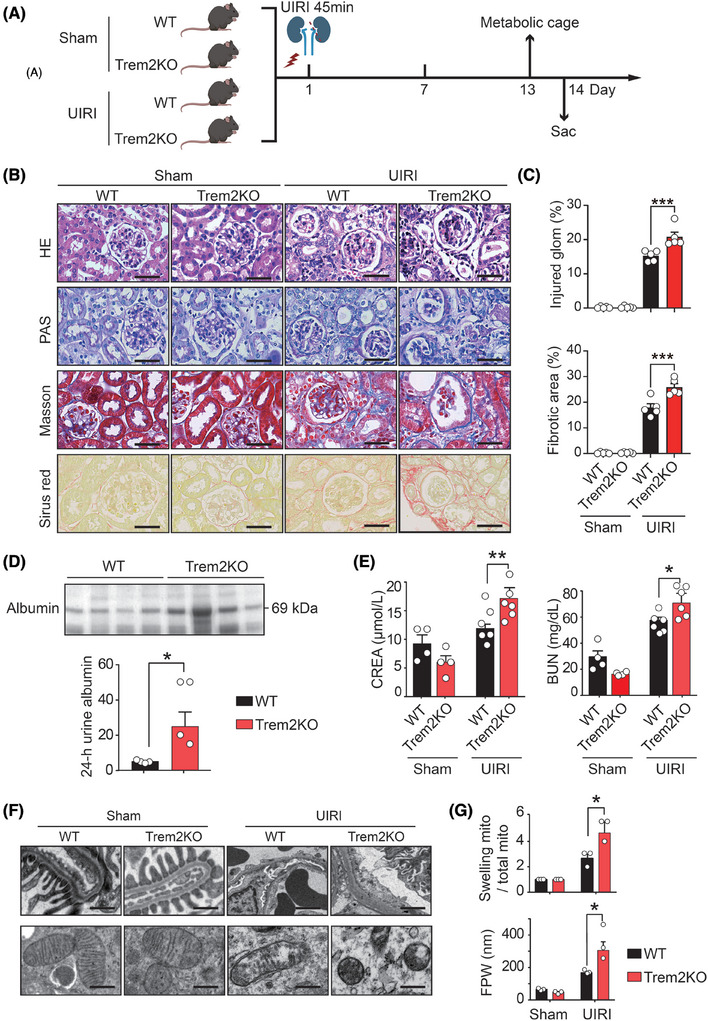
Trem2KO mice exhibited aggravated kidney injury and fibrosis after unilateral ischaemia–reperfusion injury (UIRI). (A) Schematic of the experimental design. (B) Representative histological images of kidney tissues from sham‐ and UIRI‐treated wild‐type (WT) and Trem2KO mice on day 14 after surgery stained with haematoxylin and eosin (H&E), PAS, Masson and Sirius Red. *n* = 5 per group. Scale bar, 50 µm. (C) Pathological evaluation of the levels of kidney injury and fibrosis from (B). *n* = 5 per group. Mean ± standard error of the mean (SEM). ***p < .001. (D) Coomassie Brilliant Blue staining results of the 24‐h urine albumin levels from UIRI‐treated WT and Trem2KO mice. *n* = 4 per group. Mean ± SEM. *p < .05. (E) Quantification of the serum creatinine (CREA) and blood urea nitrogen (BUN) levels in the four groups. Sham WT and Trem2KO groups: *n* = 4; UIRI WT and Trem2KO groups: *n* = 6. Mean ± SEM. *p < .05, **p < .01. (F) Transmission electron microscope (TEM) images showing the structures of the foot process in podocytes and the mitochondria in tubular cells. Scale bar, 500 nm. (G) Statistical analysis of the average width of foot processes (FPW) and the number of swollen mitochondria in tubular cells from the four groups. *n* = 3 per group. Mean ± SEM. *p < .05.

To investigate the contribution of Trem2 macrophages to the AKI–CKD transition, WT and Trem2 knockout (Trem2KO) mice were subjected to renal UIRI (Figure 1A), which has been shown to be a reliable and robust animal model for studying the transition from AKI to CKD.^45^ H&E and PAS staining of kidney sections from WT UIRI mice on day 14 after UIRI revealed several pathological features of CKD, including tubular dilatation, tubular atrophy, tubular necrosis and loss of tubular borders. Masson's trichrome and Sirius Red staining revealed that the kidneys of the WT UIRI mice exhibited interstitial fibrosis compared with those of the WT sham mice (Figure 1B). Surprisingly, compared with WT mice, Trem2KO mice presented exacerbated renal injury and fibrosis after UIRI, including increased levels of tubular atrophy, dilatation and interstitial fibrosis (Figure 1C). In addition, Trem2KO mice presented increased levels of urine albumin (Figure 1D), serum creatinine and BUN (Figure 1E) on day 14 post‐UIRI, suggesting that TREM2 played a protective role in the AKI–CKD transition. To further evaluate the effect of TREM2 on glomerular podocytes and tubular cells, we analysed their ultrastructures via TEM. The TEM images revealed no difference between the podocyte foot process and the tubular cell mitochondrial structures of the WT and Trem2KO sham mice. On day 14 after UIRI, WT mice presented marked foot process effacement (FPE) in podocytes and mitochondrial swelling in tubular epithelial cells. Notably, Trem2KO mice presented significantly increased levels of FPE and mitochondrial swelling on day 14 after UIRI (Figure 1F,G). Taken together, these findings suggested that knockout of Trem2 exacerbated renal injury and fibrosis after UIRI in animals.

### 3.3 Trem2 deficiency increases the levels of renal inflammatory cytokines and fibrosis after UIRI

**FIGURE 2:**
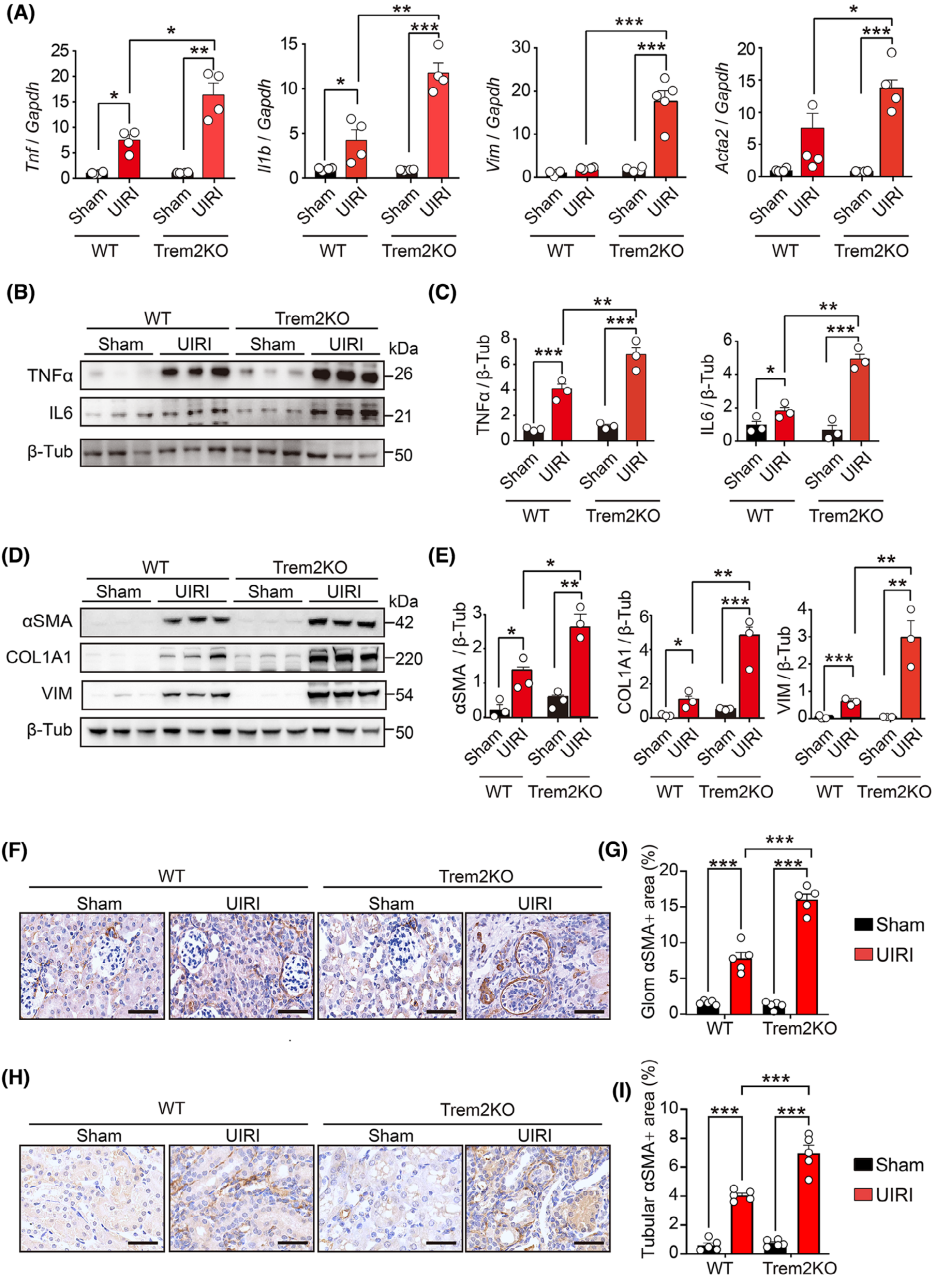
Knockout of Triggering Receptor Expressed on Myeloid Cells 2 (TREM2) increased macrophage‐mediated inflammation and kidney fibrosis in acute kidney injury–chronic kidney disease (AKI–CKD) transition mice. (A) Quantitative polymerase chain reaction (qPCR) results of *Tnf*, *Il1b*, *Vim* and *Acta2* mRNA levels in kidneys from sham‐ and unilateral ischaemia–reperfusion injury (UIRI)‐treated wild‐type (WT) and Trem2KO mice on day 14. *n* > 3 per group. Mean ± standard error of the mean (SEM). *p < .05. (B, C) Western blotting images and quantification results of tumour necrosis factor‐α (TNF‐α) and interleukin 6 (IL6) protein levels in kidneys from sham‐ and UIRI‐treated WT and Trem2KO mice on day 14. *n* = 3 per group. Mean ± SEM. **p < .01, ***p < .001. (D, E) Western blotting images and quantification results of alpha smooth muscle actin (αSMA), collagen type 1 alpha 1 (COL1A1) and vimentin (VIM) protein levels in kidneys from sham‐ and UIRI‐treated WT and Trem2KO mice on day 14. *n* = 3 per group. Mean ± SEM. *p < .05, **p < .01, ***p < .001. (F, G) Immunohistochemical (IHC) staining images and quantification results of αSMA expression in the glomerular regions from the four groups. *n* = 5 per group. Scale bar, 50 µm. Mean ± SEM. ***p < .001. (H, I) IHC staining images and quantification results of αSMA expression in the tubulointerstitial regions from the four groups. *n* = 5 per group. Scale bar, 50 µm. Mean ± SEM. ***p < .001.

Previous studies have reported that renal fibrosis is a hallmark of CKD, and its level is closely associated with the risk of progression to ESRD.^46^ Tubular epithelial cells and fibroblasts are at the centre of renal fibrosis, with inflammation serving as a crucial driver of its progression, which is mediated by diverse cytokines from immune cells. Key cytokines include transforming growth factor‐β (TGF‐β), and tumour necrosis factor‐α (TNF‐α) and various interleukins (ILs).^47^, ^48^ To assess renal injury and fibrosis progression, we quantified the mRNA levels of *Tnf*, *Il1b*, *Acta2* and *Vim* in the kidneys of sham‐ and UIRI‐operated WT and Trem2KO mice on day 14 post‐UIRI. Notably, the levels of these pro‐inflammatory cytokines and fibrotic markers were markedly elevated in Trem2KO mice compared with WT mice post‐UIRI (Figure 2A). Western blot analysis confirmed that Trem2 deficiency increased the expression of pro‐inflammatory cytokines (Figure 2B,C) and fibrotic markers (Figure 2D,E) post‐UIRI. Immunohistochemical staining further revealed increased αSMA expression in glomerular and tubular regions from both WT and Trem2KO mice post‐UIRI, with Trem2KO mice exhibiting a more pronounced increase than WT mice (Figure 2F–I). Collectively, these data implicated Trem2 as a pivotal factor in mitigating pathological changes during the AKI–CKD transition in mice.

### 3.4 Trem2 expression is upregulated and associated with immune cell infiltration in the AKI–CKD transition mice

**FIGURE 3:**
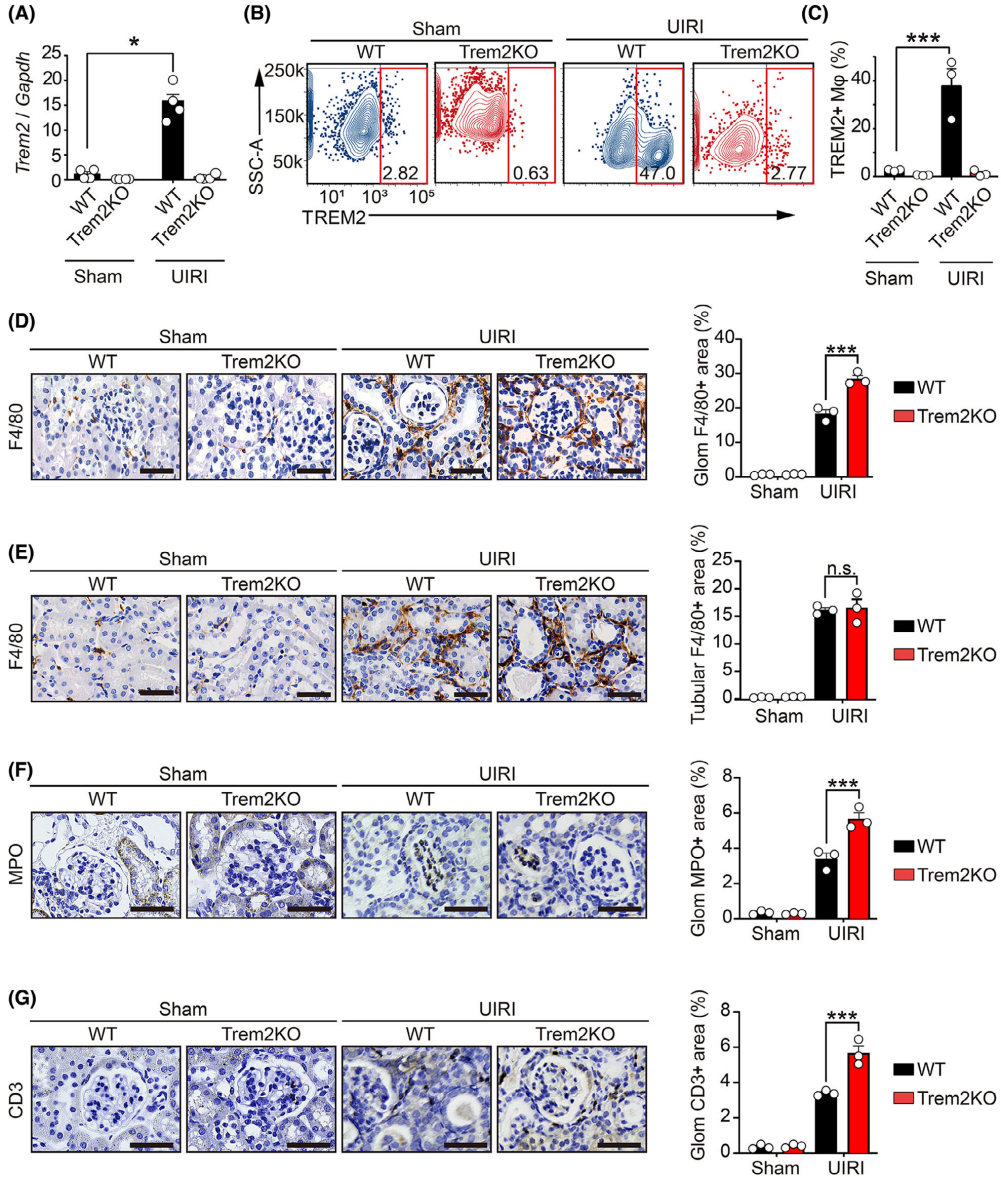
Upregulation of Triggering Receptor Expressed on Myeloid Cells 2 (TREM2) expression in renal macrophages from acute kidney injury–chronic kidney disease (AKI–CKD) transition mice. (A) Quantitative polymerase chain reaction (qPCR) results of *Trem2* mRNA levels in kidneys from sham‐ and unilateral ischaemia–reperfusion injury (UIRI)‐treated wild‐type (WT) and Trem2KO mice on day 14 post‐UIRI. *n* = 4 per group. Mean ± standard error of the mean (SEM). *p < .05. (B, C) Flow cytometry analysis of the F4/80+TREM2+ macrophage percentages in kidneys from four groups on day 14 post‐UIR. *n* = 3 per group. Mean ± SEM. ***p < .001. (D, E) Immunohistochemical (IHC) staining images and quantification results of F4/80+ macrophages in the glomerular (D) and tubular (E) regions of kidneys from four groups. *n* = 3 per group. Scale bar 50 µm. Mean ± SEM. ***p < .001. (F, G) IHC staining images and quantification results of MPO+ neutrophils (F) and CD3+ T cells (G) in the glomerular regions of kidneys from four groups. *n* = 3 per group. Scale bar 50 µm. Mean ± SEM. ***p < .001.

Given that Trem2 is selectively expressed on myeloid cells, including monocytes and macrophages, we explored its role in macrophages during the AKI–CKD transition. We first investigated the mRNA expression of inflammatory cytokines (*Tnf* and *Il6*) and kidney injury markers (*Kim*‐1 and *Ngal*) in WT and Trem2KO mice on day 3 post‐UIRI. The results showed that Trem2KO mice exhibited increased level of *Ngal*, suggesting an increased level of kidney injury in these mice (Figure S2A). However, the numbers of F4/80+ macrophages, MPO+ neutrophils and CD3+ T cells were not affected in Trem2KO mice compared to that of WT mice on day 3 post‐UIRI (Figure S2B–D). Notably, *Trem2* mRNA levels were significantly elevated in the kidneys of UIRI‐operated WT mice compared with the sham‐operated controls on day 14. Conversely, *Trem2* mRNA was undetectable in both the sham‐ and UIRI‐operated Trem2KO mice (Figure 3A). To further elucidate the changes in macrophages in the kidneys of WT and Trem2KO mice, we isolated renal macrophages using an anti‐F4/80 microbead kit and performed flow cytometry with TREM2 and CD11b antibodies. Consistent with the mRNA findings, flow cytometry revealed significant upregulation of TREM2 in renal macrophages from WT mice post‐UIRI, whereas no TREM2 expression was detected in Trem2KO macrophages (Figure 3B,C). Surprisingly, CD11b analysis revealed an increase in total renal macrophages (CD11b+F4/80+) in both WT and Trem2KO mice post‐UIRI, with no significant difference between the two genotypes (Figure S3A). Intriguingly, immunohistochemical staining revealed a significant increase in periglomerular F4/80+ macrophages, but not tubulointerstitial macrophages, in Trem2KO mice compared with WT mice post‐UIRI (Figure 3D,E). In addition, immunohistochemistry results indicated increased levels of neutrophil and T cell infiltration in kidneys of Trem2KO mice compared to that of WT mice on day 14 post‐UIRI (Figure 3F,G). These findings suggested that Trem2 knockout did not affect the total number of F4/80+ macrophages but did alter their population and distribution, and affected immune cell infiltration during AKI–CKD transition. Given that Trem2 deficiency exacerbates renal injury and fibrosis, Trem2 may confer renal protection by modulating macrophage‐mediated functions post‐UIRI.

### 3.5 Trem2 was upregulated upon hypoxia‐reoxygenation and regulated macrophage cytokine expression and phagocytosis

**FIGURE 4:**
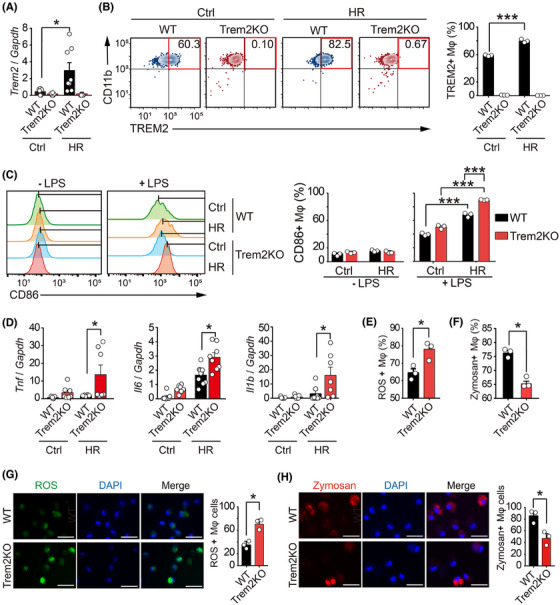
Triggering Receptor Expressed on Myeloid Cells 2 (TREM2) was upregulated by hypoxia‐reoxygenation (HR) via HIF‐1α and regulated macrophage inflammation and phagocytosis. (A) Quantitative polymerase chain reaction (qPCR) results of the *Trem2* mRNA levels in wild‐type (WT) and Trem2KO bone marrow‐derived macrophages (BMDMs) treated with normoxia (Ctrl) and HR. *n* = 8 per group. Mean ± standard error of the mean (SEM). *p < .05. (B) Flow cytometry analysis of the TREM2 expression in WT and Trem2KO BMDMs treated with normoxia (Ctrl) and HR. *n* = 3 per group. Mean ± SEM. *p < .05. (C, D) Flow cytometry of the CD86 expression on polarised and unpolarised WT and Trem2KO BMDMs treated with normoxia (Ctrl) and HR with and without lipopolysaccharide (LPS) pre‐treatment. *n* = 3 per group. Mean ± SEM. *p < .05, **p < .01. (D) qPCR results of mRNA levels of the pro‐inflammatory cytokines in WT and Trem2KO BMDMs treated with normoxia (Ctrl) and HR. *n* > 5 per group. Mean ± SEM. *p < .05. (E, F) Flow cytometry analysis results of the ROS (E) and Zymosan phagocytic (F) levels in WT and Trem2KO BMDMs after HR treatment. *n* = 3 per group. Mean ± SEM. *p < .05. (G, H) Immunofluorescence analysis of the ROS (G) and Zymosan phagocytic (H) levels in WT and Trem2KO BMDMs after the HR treatment. *n* = 3 per group. Mean ± SEM. *p < .05.

Although we found that Trem2 expression was increased in the renal macrophages of UIRI mice, the mechanism by which Trem2 was upregulated remains unclear. Hypoxia has been shown to play important roles in AKI and renal fibrosis. To explore whether hypoxia controlled the Trem2 expression, we treated WT and Trem2KO BMDMs with hypoxia‐reoxygenation (HR) stimulation, which mimicked the hypoxic conditions of UIRI mice. As a result, Trem2 mRNA levels were increased in HR‐treated BMDMs than in control BMDMs (Figure 4A). Flow cytometry analysis further confirmed the increased expression of TREM2 at the protein level in HR‐treated WT BMDMs compared with control BMDMs (Figure 4B). Neither the mRNA nor the protein of Trem2 was detected in Trem2KO macrophages with or without HR stimulation. These results suggested that hypoxia‐oxygenation stimulation could regulate Trem2 expression in macrophages. Indeed, several studies have shown that hypoxia can regulate the inflammatory phenotype of macrophages, including the upregulation of genes related to macrophage survival and activation. We therefore investigated the effect of Trem2 on the phenotypic changes in macrophages upon HR stimulation. Flow cytometry analysis revealed that in the absence of LPS polarisation, the expression level of CD86, a macrophage activation marker, remained unchanged in both WT and Trem2KO BMDMs with and without HR stimulation. However, after LPS polarisation, the expression level of CD86 was significantly greater in HR‐treated Trem2KO BMDMs than in HR‐treated WT BMDMs (Figure 4C). In addition, the mRNA expression levels of pro‐inflammatory cytokines, including *Tnf*, *Il6* and *Il1b*, were significantly greater in HR‐treated Trem2KO BMDMs than in HR‐treated WT BMDMs (Figure 4D). These findings demonstrated that knockout of Trem2 increased macrophage activation and pro‐inflammatory cytokine expression, suggesting that Trem2 might negatively regulate macrophage activation and inflammation.

It has been reported that TREM2 promotes the phagocytosis of *Mycobacterium tuberculosis* and blocks the production of TNF‐α, IL‐1β and ROS while enhancing the generation of interferon‐β (IFN‐β) and IL‐10. We therefore investigated the differences in reactive oxygen species (ROS) production and phagocytosis between WT and Trem2KO BMDMs. Using the ROS fluorescent probe and Zymosan phagocytosis kit, flow cytometry analysis confirmed that knockout of Trem2 increased the ROS production and decreased the Zymosan phagocytotic level in macrophages upon HR stimulation (Figures 4E,F and S3B,C). In addition, fluorescence images further confirmed that knockout of Trem2 increased the ROS production and decreased the Zymosan phagocytotic level in BMDMs upon HR treatment (Figure 4G,H). Interestingly, the upregulation effect of HR on *Trem2* mRNA expression in BMDMs was abolished by a HIF‐1α inhibitor LW6, suggesting that Trem2 gene expression is regulated by hypoxia via HIF‐1α (Figure 3D). These results indicated that Trem2 could be upregulated by HR, which could in turn regulate macrophage cytokine expression and phagocytosis, eventually protecting the kidney from the AKI–CKD transition.

### 3.6 RNA‐seq analysis of the transcriptomic changes in Trem2KO macrophages

**FIGURE 5:**
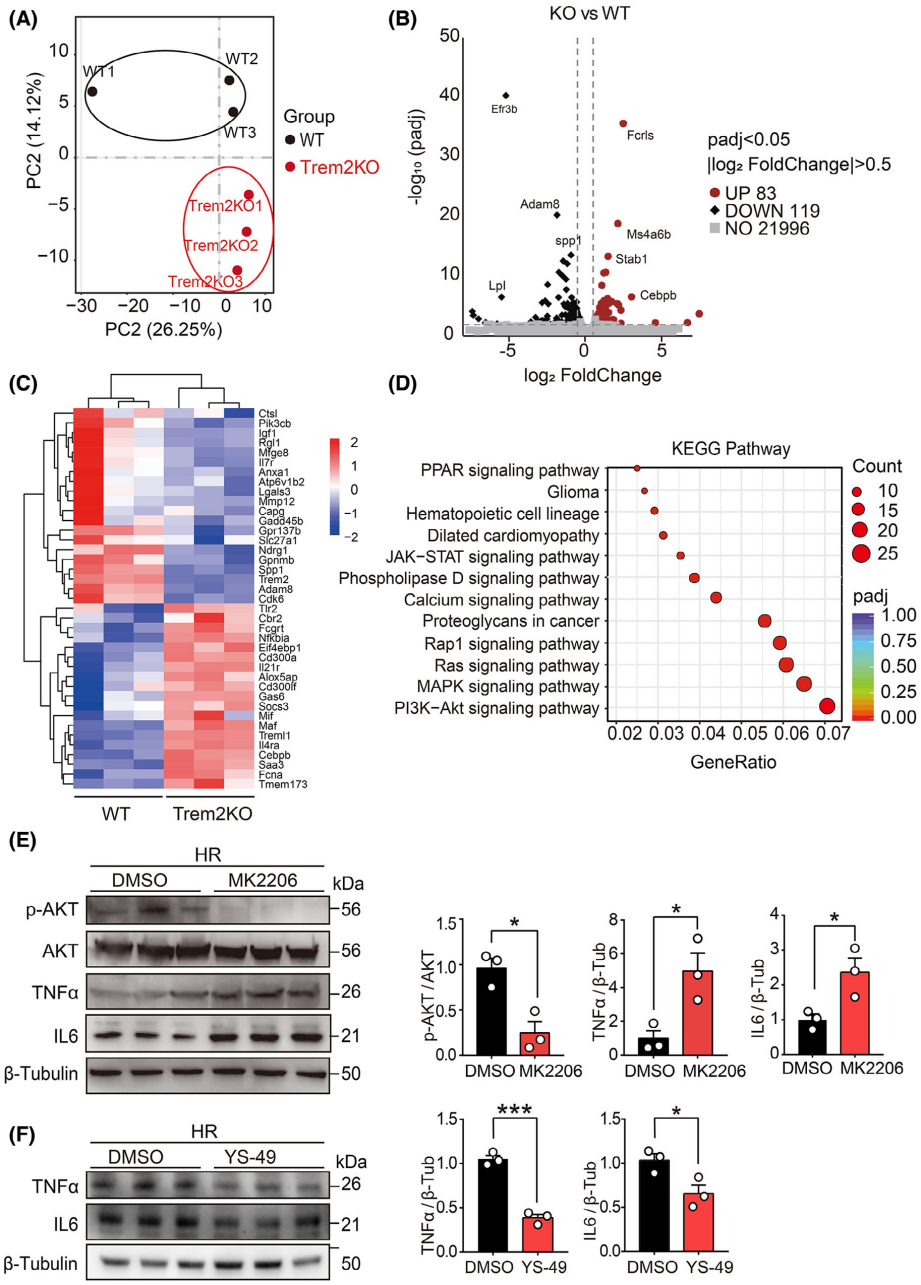
RNA‐seq analysis of the transcriptomic changes in Trem2KO macrophages. (A) PCA result showing the wild‐type (WT) and Trem2KO bone marrow‐derived macrophages (BMDMs) after the hypoxia‐reoxygenation (HR) treatment for 24 h. Each group contained three biological replicates. (B) Volcano plot of the differentially expressed genes (DEGs) between two groups. 83 and 119 genes were found to be upregulated and downregulated, respectively. (C) Heatmap showing the normalised expression of DEGs in two groups. (D) KEGG pathway enrichment analysis of the downregulated DEGs in Trem2KO BMDMs. Dot size represents the number of genes in each KEGG pathway. Colour represents the adjusted *p*‐value (*p*‐adj). (E) Western blotting results of p‐AKT, AKT, tumour necrosis factor‐α (TNF‐α) and interleukin 6 (IL6) protein levels in WT BMDMs treated with HR in the presence and absence of a p‐AKT inhibitor MK2206 for 24 h. *n* = 3 per group. Mean ± standard error of the mean (SEM). *p < .05. (F) Western blotting results of TNFα and IL6 protein levels in WT BMDMs treated with HR in the presence and absence of a PI3K activator YS‐49 for 24 h. *n* = 3 per group. Mean ± SEM. *p < .05.

To further understand the effect of Trem2 on transcriptomic changes in macrophages, we used RNA‐seq to analyse the transcriptomic profiles of WT and Trem2KO BMDMs. PCA was used to compare the transcriptomic orthogonal variables between the two groups (Figure 5A). A volcano plot revealed that 83 and 119 genes were upregulated and downregulated, respectively, in Trem2KO BMDMs compared with WT BMDMs (Figure 5B). A heatmap was generated to visualise the genes that were differentially expressed between the two groups (Figure 5C). KEGG enrichment analysis was conducted on these DEGs. The results revealed 12 enriched KEGG pathways, among which PI3K‐AKT, MAPK and Ras signalling were the top 3 enriched pathways (Figure 5D). According to previous reports, Trem2 activation in microglia reduces neuroinflammation and improves cognitive dysfunction in mice through the PI3K‐AKT pathway. Moreover, in mice with LPS‐induced neural damage, inhibition of PI3K‐AKT abolished the protective effect of Trem2.^49^ These results suggested that Trem2 might regulate the inflammatory and phagocytotic phenotypes of macrophages via the PI3K‐AKT pathway. To validate this pathway, we examined phospho‐AKT and total AKT protein levels in WT and Trem2KO BMDMs after HR stimulation. The results suggested that p‐AKT was significantly lower in Trem2KO BMDMs than in WT BMDMs, which was consistent with our RNA‐seq results. Moreover, the TNFα level was significantly increased in Trem2KO BMDMs, which was consistent with our in vitro and in vivo results (Figure S4A). To further validate the contribution of PI3K‐AKT in macrophages, WT BMDMs were treated with the p‐AKT inhibitor MK2206 prior to HR stimulation. Pre‐treatment with the p‐AKT inhibitor MK2206 significantly reduced the p‐AKT level in WT BMDMs, and more interestingly, the level of TNFα was increased in WT BMDMs pre‐treated with MK2206 (Figure 5E). In addition, treatment with a PI3K activator YS‐49 could decrease the TNFα and IL6 expression but increase the phagocytic level in BMDMs (Figures 5F and Figure S4B). Taken together, these results suggested that Trem2 could regulate macrophage inflammation and phagocytosis through the PI3K‒AKT signalling pathway.

### 3.7 Macrophage Trem2 knockout leads to tubular epithelial cell injury and fibrosis

We showed that Trem2 may regulate macrophage inflammation and phagocytosis. However, it remained unclear whether these regulatory effects of Trem2 were involved in renal cell injury and fibrosis. Renal tubular epithelial cells (RTECs), among the most abundant cells in the renal parenchyma, are critical for renal physiology. Their dysfunction and damage have been shown to contribute to kidney injury and fibrosis.^50^ To investigate the effects of Trem2 macrophages on tubular cells, primary mouse RTECs (mRTECs) were isolated and cultured with the conditioned medium (CM) from WT or Trem2KO BMDMs upon HR stimulation (Figure S5A). As expected, compared with mRTECs cultured with CM from WT BMDMs, those cultured with CM from Trem2KO BMDMs presented increased expression levels of fibrotic genes, including Vim and Acta2, at the mRNA level. Interestingly, cells cultured with CM from WT BMDMs treated with the phospho‐AKT inhibitor MK2206 also presented increased expression levels of Vim and Acta2, suggesting that the PI3K‐AKT pathway was downstream of Trem2 in macrophages (Figure S5B).

Consistent with the mRNA results, the immunofluorescence staining results further confirmed that mRTECs cultured with CM from Trem2KO BMDMs presented higher immunofluorescent levels of fibronectin (FN) and type 1 collagen (COL1A1) than did those cultured with CM from WT BMDMs. In addition, the immunofluorescence results revealed that cells cultured with CM from WT BMDMs treated with the phospho‐AKT inhibitor MK2206 presented increased levels of FN and COL1A1 (Figure S5C–F), which further supported the notion that the PI3K‐AKT pathway was downstream of Trem2 in macrophages. As shown by the flow cytometry results, compared with WT BMDMs, Trem2KO BMDMs not only presented increased fibrotic gene expression in tubular epithelial cells but also exhibited increased levels of apoptosis. Moreover, treatment of WT BMDMs with the phospho‐AKT inhibitor MK2206 also significantly increased the apoptotic level of tubular epithelial cells (Figure S5G,H). These results suggested that Trem2 knockout in macrophages led to increased levels of tubular epithelial cell injury and fibrosis.

### 3.8 Adoptive cell therapy with TREM2‐overexpressing BMDMs mitigates the AKI–CKD transition

**FIGURE 6:**
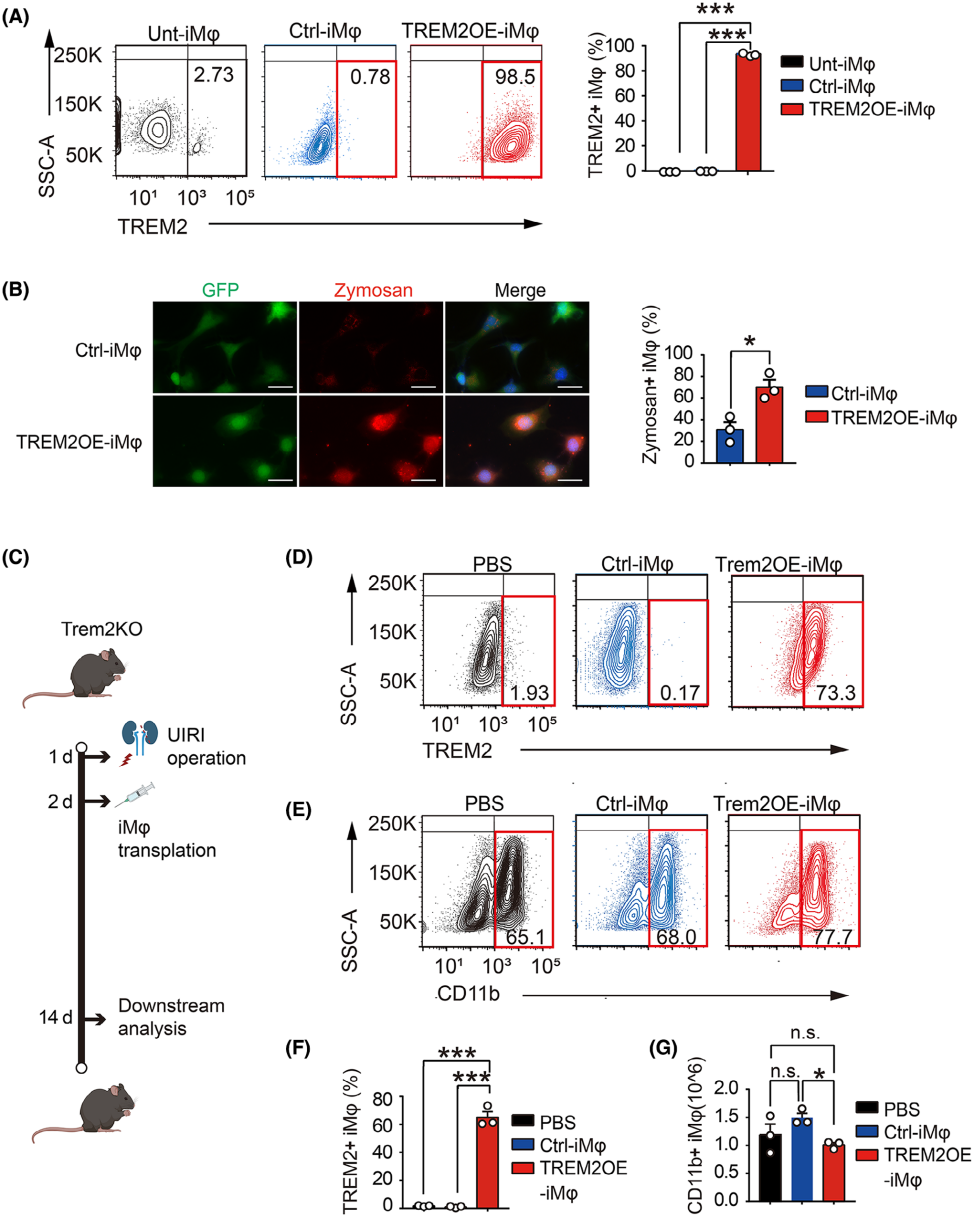
Developing an adoptive cell therapy using Triggering Receptor Expressed on Myeloid Cells 2 (TREM2)‐overexpressing bone marrow‐derived macrophages (BMDMs) to unilateral ischaemia–reperfusion injury (UIRI) mice. (A) Generation and characterisation of the immortalised macrophages overexpressing TREM2 (TREM2OE‐iMφ). Flow cytometry analysis of the TREM2 expression levels in uninfected (unt), empty vector lentivirus‐infected (Ctrl) and TREM2 lentivirus‐infected (TREM2OE‐iMφ). *n* = 3 per group. Mean ± standard error of the mean (SEM). ***p < .001. (B) Evaluation of the phagocytosis levels in Ctrl‐ and Trem2OE‐iMφ using a Zymosan phagocytosis kit. Left: Fluorescent images of the phagocytosed Zymosan in Ctrl‐ and Trem2OE‐iMφ. Right: Quantified result of Zymosan‐positive macrophages in two groups. *n* = 3 per group. Mean ± SEM. *p < .05. (C) Schematic of the experimental design. TREM2KO mice were intravenously injected with PBS, Ctrl‐iMφ (1 × 10^6^) and TREM2OE‐iMφ (1 × 10^6^) on the second day after the UIRI. Mice were sacrificed on day 14 and renal macrophages were sorted with F4/80+ magnetic beads and analysed by flow cytometry. (D, E) Flow cytometry result of the percentages of TREM2+ and CD11b+ macrophages in F4/80+ sorted renal macrophages from three groups. (F) Percentages of TREM2+ macrophages in F4/80+ sorted renal macrophages from three groups. *n* = 3 per group. Mean ± SEM. ***p < .001. (G) Numbers of F4/80+CD11b+ macrophages from three groups. *n* = 3 per group. Mean ± SEM. *p < .05. n.s., not significant.

Because our data indicated that Trem2 exerted a strong renal protective effect on the AKI–CKD transition, it was of interest to determine whether adoptive cell therapy using an immortalised BMDM cell line overexpressing TREM2 (TREM2OE‐iMφ) could reduce the AKI–CKD transition. Therefore, we generated GFP control (Ctrl)‐ and TREM2OE‐iMφ using lentivirus infection. The expression of TREM2 in untransfected (Unt)‐, GFP control (Ctrl)‐ and TREM2OE‐iMφ was tested using flow cytometry. The results revealed that more than 98% of macrophages expressed high levels of TREM2 in TREM2OE‐iMφ but not in Unt‐ or Ctrl‐iMφ (Figure 6A). We then examined whether TREM2 overexpression could regulate the phagocytosis of iBMDMs via a zymosan phagocytosis assay. Surprisingly, overexpression of TREM2 in iBMDMs significantly increased the level of phagocytosis of zymosan (Figure 6B), which indicated that overexpression of TREM2 successfully modulated macrophage functions. To investigate the potential therapeutic benefits of TREM2OE‐iMφ in the AKI–CKD transition, we transferred two types of macrophages (GFP Ctrl‐ and TREM2OE‐iMφ) intravenously into Trem2KO mice 2 days post‐UIRI. PBS‐treated mice served as a baseline. Kidney tissues and renal macrophages were then collected on day 14 for downstream analysis (Figure 6C). Compared with the Ctrl‐iMφ, the transfer of TREM2OE‐iMφ significantly increased the percentage of TREM2+ macrophages in F4/80+CD11b+ macrophages in the kidney at 14 days post‐UIRI (Figure 6D–F). These results suggested that TREM2OE‐iMφ had been successfully transferred into the kidneys of Trem2KO mice post‐UIRI. Interestingly, the total number of F4/80+CD11b+ macrophages was decreased in the kidneys transferred with Trem2OE‐iMφ (Figure 6G), which may be due to the reduced inflammatory environment and decreased immune cell infiltration level by TREM2OE‐iMφ.

**FIGURE 7:**
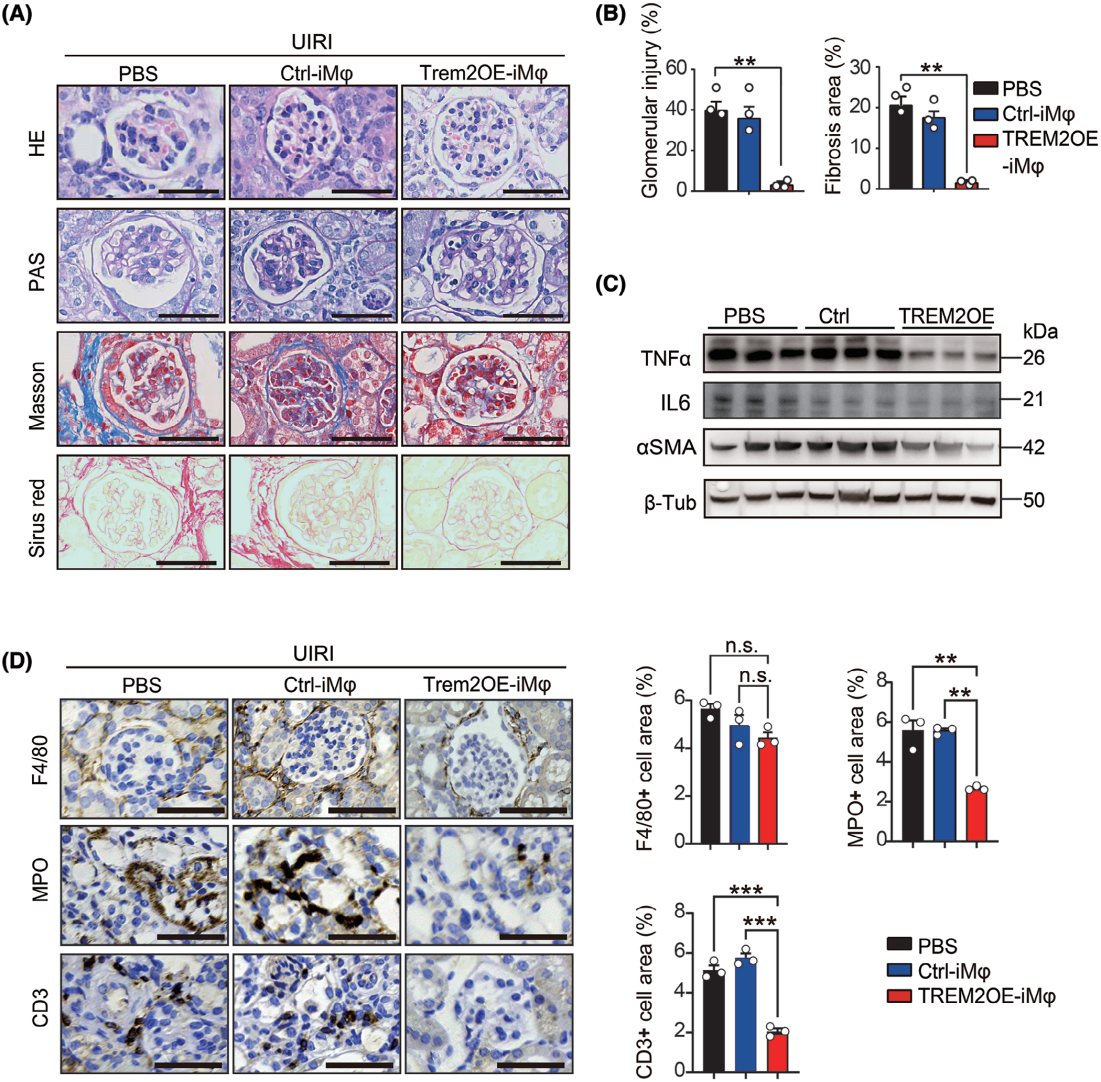
Transfer of Triggering Receptor Expressed on Myeloid Cells 2 (TREM2)‐overexpressing bone marrow‐derived macrophages (BMDMs) mitigated the acute kidney injury–chronic kidney disease (AKI–CKD) transition in unilateral ischaemia–reperfusion injury (UIRI) mice. (A) Representative histological images of kidney tissues stained with haematoxylin and eosin (H&E), PAS, Masson and Sirius Red from TREM2KO mice administrated with PBS, Ctrl‐ and Trem2OE‐iMφ on day 14 post‐UIRI. *n* = 5 per group. Scale bar 50 µm. (B) Analysis results of the glomerular injury and fibrosis area from (A). *n* = 5 per group. Mean ±   standard error of the mean (SEM). **p < .01. (C) Western blotting results of tumour necrosis factor‐α (TNF‐α) and αSMA protein levels in kidneys from three groups on day 14 post‐UIRI. (D) Immunohistochemical (IHC) staining images and quantification results of F4/80+ macrophages, MPO+ neutrophils and CD3+ T cells in kidneys from three groups. *n* = 3 per group. Scale bar 50 µm. Mean ± SEM. **p < .01, ***p < .001. n.s., not significant.

Histological results of H&E, PAS, Masson and Sirius Red staining revealed that after UIRI, Trem2KO mice treated with PBS and transferred with the GFP Ctrl‐iMφ presented similar pathological features to CKD, including glomerular atrophy, glomerular sclerosis and tubulointerstitial fibrosis (Figure 7A). However, the transfer of Trem2OE‐iMφ significantly reduced both the degree of glomerular injury and the degree of tubulointerstitial fibrosis in Trem2KO mice post‐UIRI (Figure 7B). In addition, Western blotting results further confirmed that the transfer of Trem2OE‐iMφ could decrease the expression of the pro‐inflammatory cytokine TNFα, IL6 and the fibrotic gene αSMA in mouse kidneys post‐UIRI (Figure 7C). In addition, transfer of Trem2OE‐iMφ could decrease the levels of neutrophil and T cell infiltration in mouse kidneys post‐UIRI (Figure 7D). Taken together, our findings suggested that an TREM2‐overexpressing macrophage‐based adoptive cell therapy holds strong translational potential in the treatment of AKI–CKD transition.

## 4 DISCUSSION

The transition from AKI to CKD is a complex process involving multiple cellular and molecular pathways, with macrophages playing a pivotal role.^51^, ^52^ Macrophages are central to the immune response and tissue remodelling in renal disease.^53^ In the context of AKI, macrophages can adopt different activation states, influencing the outcome of injury and repair processes. Depletion of macrophages via different techniques revealed the pathogenic role of pro‐inflammatory macrophages in both AKI and CKD. The depletion of macrophages by liposomal clodronate at the early stage significantly has been shown to attenuate renal injury and subsequent fibrosis in IRI and rhabdomyolysis mice.^54^, ^55^ However, by producing anti‐inflammatory factors and phagocytosing cell debris, macrophages also have anti‐inflammatory and tissue repair effects.^56^ For example, through their anti‐inflammatory and wound healing functions, Ly6C^int^ macrophages have been shown to contribute to kidney repair.^57^ Depleting macrophages in the late stage resulted in decreased tubular epithelial cell proliferation and renal repair in IRI mice.^58^, ^59^, ^60^ These findings highlight the complexity of macrophages in kidney disease onset and progression.

In this study, to explore the single‐cell transcriptomic changes in macrophages during the transition from AKI to CKD, we first analysed a scRNA‐seq dataset from different time points in UIRI mice. Consistent with many previous studies, the scRNA‐seq results confirmed that both macrophage populations and transcriptomic profiles significantly changed during the AKI–CKD transition. Interestingly, after DEG analysis, we discovered that Trem2, which was highly expressed within those cell clusters that were selectively expanded during the transition, was significantly upregulated during the AKI–CKD transition. In addition, using a UIRI mouse model, we confirmed that TREM2 expression was upregulated at the mRNA and protein level during the AKI–CKD transition. These results suggested that TREM2 macrophages might be actively involved in the AKI–CKD transition. However, their specific role in this process remains unclear.

TREM2, a receptor that is highly expressed on macrophages,^24^ has emerged as a key regulator of macrophage phenotypes and functions, including inflammatory responses and phagocytosis. Using Trem2‐deficient mice, we showed that knockout of Trem2 mitigated renal macrophages from properly regulating renal inflammation, which led to increased renal fibrosis in vivo. Interestingly, while Trem2 knockout did not affect the quantity of renal macrophages, it did affect their renal spatial localisation and functions, further suggesting that TREM2 served as an important regulator of renal macrophages in the AKI–CKD transition. Indeed, after HR stimulation, Trem2KO macrophages presented increased levels of pro‐inflammatory cytokines and decreased levels of phagocytosis in vitro. In addition, mouse tubular epithelial cells cultured with conditioned medium from Trem2KO macrophages presented increased expression levels of fibrotic genes, which was consistent with the in vivo results. Using RNA‐seq analysis, we elucidated that the PI3K‐AKT pathway was the downstream signalling pathway of Trem2 in renal macrophages. Knockout of Trem2 resulted in a decreased level of phosphorylated AKT, which has a multifunctional role in regulating inflammation and apoptosis. Pharmacological inhibition of phosphorylated AKT also increased the level of TNFα. These results suggested that TREM2 was a key regulator of macrophage inflammation and phagocytosis via PI3K‐AKT pathway, which could mitigate the transition from AKI to CKD. However, other macrophage receptors or signalling pathways (such as those involving the NF‐κB or MAPK pathways) may compensate for the loss of TREM2, thereby modulating inflammation, fibrosis and tissue repair processes in this disease condition.

In general, cell therapy refers to the transfer of living cells into the body as a medical treatment. The first cell therapy in the history of modern medicine dates back to the 1900s, when the intravenous infusion of whole blood was performed. Recently, macrophage‐based cell therapies have attracted increasing interest among researchers and clinicians because of their critical role in health and disease,^61^, ^62^, ^63^, ^64^, ^65^ including fighting tumours, eliminating pathogens and repairing injuries.^66^, ^67^ To date, several phase II and III clinical trials have used macrophages for the treatment of diseases such as cardiomyopathy, limb ischaemia, stroke and chronic anal fissure, suggesting that macrophage‐based cell therapies hold great clinical potential in regenerative medicine.^68^ In this study, we explored the translational potential of macrophages overexpressing TREM2 in the AKI–CKD transition. Using macrophages overexpressing TREM2, we demonstrated that the transfer of a single dose of these macrophages in UIRI mice successfully reduced renal inflammation, glomerular damage and tubulointerstitial fibrosis, which are the hallmarks of CKD. This is the first study to demonstrate the translational potential of TREM2‐overexpressing macrophage‐based cell therapy in the treatment of kidney disease. Given that TREM2 macrophages have been shown to play a protective role in a wide range of diseases, we believe that our study provides a novel approach for the treatment of a variety of diseases.

### 4.1 Limitations

Our current study has several limitations. First, the overexpression of TREM2 may not fully recapitulate the physiological conditions under which TREM2 is naturally expressed in macrophages. Immortalised cell lines, such as the iBMDMs used in this study, may exhibit altered signalling pathways or a heightened response to stimuli, which may not accurately reflect the behaviour of primary macrophages in vivo. Additionally, the levels of TREM2 overexpression in the iBMDMs may exceed those observed in the context of injury or disease, potentially resulting in exaggerated responses. Future studies employing more physiologically relevant models, such as primary macrophages or inducible overexpression systems, will help to confirm the translational relevance of these findings. While our study focused on the renal effects of TREM2‐overexpressing macrophages, we acknowledge that the transfer of TREM2OE‐iMφ could have systemic effects that influence other tissues during injury or health. While global TREM2 KO mice provide valuable insights into the overall impact of TREM2 deficiency, they do not allow for the precise dissection of tissue‐specific roles, especially in macrophages. Future experiments using macrophage‐specific TREM2 knockout mice would provide a more focused understanding of the macrophage‐specific contributions to the progression of AKI to CKD.

In conclusion, our study not only provides mechanistic insights into the role of Trem2 in regulating macrophage‐mediated inflammation and fibrosis in kidney injury but also opens a new avenue for macrophage‐based cell therapy to treat kidney diseases.

## AUTHOR CONTRIBUTIONS

Yiming Zhou, Chun Tang and Xiaohua Wang generated the hypotheses and designed the experiments. Yiming Zhou and Chun Tang funded this study. Yating Zhang conducted all the experiments. Yu Liu and Hongyu Li participated in the in vivo experiments. Hanzhi Liang, Chipeng Guo, Yufei Du and Le Wang participated in the in vitro experiments. Siweier Luo conducted the single‐cell RNA‐seq analysis and generated the data. Yiming Zhou and Yating Zhang generated the figures and wrote the initial manuscript. All the authors participated in manuscript writing and agreed to publish this manuscript.

## CONFLICT OF INTEREST STATEMENT

The authors declare no conflicts of interest.

## ETHICS STATEMENT

The animal protocols were in accordance with the NIH Guidebook for the Care and Use of Laboratory Animals and approved by the Ethics Committee of Sun Yat‐sen Memorial Hospital (No. AP20230083).

## Supporting information



Supporting Information

Supporting Information

## Data Availability

The data that support the findings of this study are available from the corresponding author upon reasonable request.
