# Supplementary material for: An adoptive cell therapy with TREM2‐overexpressing macrophages mitigates the transition from acute kidney injury to chronic kidney disease
Source: Clin Transl Med. 2025 Feb 25;15(3):e70252. doi: 10.1002/ctm2.70252 (PMC11859120; doi:10.1002/ctm2.70252)
Supplement: Supplementary file 1 — Supporting Information [file CTM2-15-e70252-s002.docx]

# Title: An adoptive cell therapy with TREM2-overexpressing macrophages mitigates the transition from acute kidney injury to chronic kidney disease.

Yating Zhang^1,2,5^, Yu Liu^3,5^, Siweier Luo^1,2,5^, Hanzhi Liang^4^, Chipeng Guo^1,2^, Yufei Du^1,2^, Hongyu Li^4^, Le Wang^1,2^, Xiaohua Wang^3,#^, Chun Tang^3,#^, Yiming Zhou^1,2,#^

1. Basic and Translational Medical Research Center, Sun Yat-sen Memorial Hospital, Sun Yat-sen University, Guangzhou, Guangdong 510120, China.
2. Guangdong Provincial Key Laboratory of Malignant Tumor Epigenetics and Gene Regulation, Guangdong-Hong Kong Joint Laboratory for RNA Medicine, Sun Yat-sen Memorial Hospital, Sun Yat-sen University, Guangzhou, Guangdong 510120, China.
3. Department of Nephrology, The Seventh Affiliated Hospital of Sun Yat-sen University, Sun Yat-sen University, Shenzhen, Guangdong, 518107, China.
4. Department of Nephrology, The First Affiliated Hospital, Sun Yat-Sen University, Guangzhou, Guangdong, 510080, China.
5. These authors contributed equally

#Corresponding authors: Yiming Zhou, Chun Tang, Xiaohua Wang.

**Supplementary Information**

**Supplementary materials and methods**

# Data availability. Raw data were obtained from the ArrayExpress database (http://www.ebi.ac.uk/arrayexpress) via the accession number E-MTAB-8002.

# Description of IRI dataset.

To investigate the dynamics of the Trem2 gene in the mouse ischemia-reperfusion injury (IRI) model, we utilized a single-cell RNA sequencing dataset published by Fernanda Duraes et al. This raw dataset is publicly available through the ArrayExpress database (accession number: E-MTAB-8002). The dataset was generated using tissue samples from a sham group and fibrotic mice at various time points post-injury, enabling the comparison of gene expression profiles across different stages of fibrosis and injury.

# Quality control and data filtering.

Single-cell RNA sequencing data were analyzed using R (v4.0.3) and Seurat (v4.0.2). The raw expression matrix was first converted into a Seurat object for further analysis. Quality control procedures included the filtering of cells based on the following criteria: (1) unique molecular identifiers (UMIs) between 200 and 3,000; (2) mitochondrial gene content less than 0.01%; and (3) ribosomal gene content less than 0.06%. Cells failing to meet these thresholds were excluded from subsequent analyses.

# De-batch Analysis and Clustering

To mitigate batch effects, canonical correlation analysis (CCA) was performed using the IntegrateData() function, with default parameters applied to preserve biological variability between samples. Samples from each group were assumed to belong to the same batch for the purpose of integration. After integration, dimensional reduction was performed on the counts matrix using PCA, retaining the first 30 principal components. To visualize and cluster the data, we utilized uniform manifold approximation and projection (UMAP), with clustering performed using the FindClusters() function. Subsequently, macrophage subsets (Cd11b^+^ macrophages) were extracted from the clusters for further analysis. To reduce technical variability while preserving biological differences, we repeated the de-batch analysis and clustering steps specifically for the macrophage subsets. For these subsets, UMAP visualization was performed with a resolution of 0.1, and cell markers were identified using the Wilcoxon test in the FindMarkers() function. The following thresholds were applied for marker identification: minimum percentage of expression (min.pct) = 0.25 and log fold change threshold (logfc.threshold) = 1. During macrophage classification, subsets with co-expression of both T cell or B cell markers and macrophage markers were excluded.

# Heatmap of Macrophage Marker Expression

To visualize the expression patterns of macrophage markers, we generated heatmaps of the top 3 most highly expressed genes in the Cd11b^+^ macrophage population and in each macrophage subpopulation after reclustering. The ComplexHeatmap R package (v2.6.2) was used for this analysis. Gene expression data were normalized and scaled across cells before visualization. The top 3 highly expressed genes in each population were selected based on their mean expression levels, and heatmaps were created to display the differential expression patterns across different clusters. Hierarchical clustering of cells was applied to reveal underlying expression relationships, with color scaling representing normalized gene expression levels.

# Cell percentage calculation

# Cell percentages were calculated through the following the steps: calculated the proportion of one kind of macrophage subset in each sample versus all cell clusters in that sample, and took the average percentage of the proportion of this type of macrophage subset in all samples of the group as the proportion of that macrophage subset in the group.

# Differentially expressed gene (DEG) analysis

# We performed differential expressed gene (DEG) analysis of the total number of macrophages using the function FindMarkers(), comparing all fibrotic and pseudofibrotic groups by the default Wilcox test.The thresholds for DEG were p-values ≤ 0.05 and logarithmic changes ≥ 0.32. After delineation of the thresholds, plots were drawn using the package EnhancedVolcano using the R package ( V1.8.0) was used to draw differential gene volcano maps.


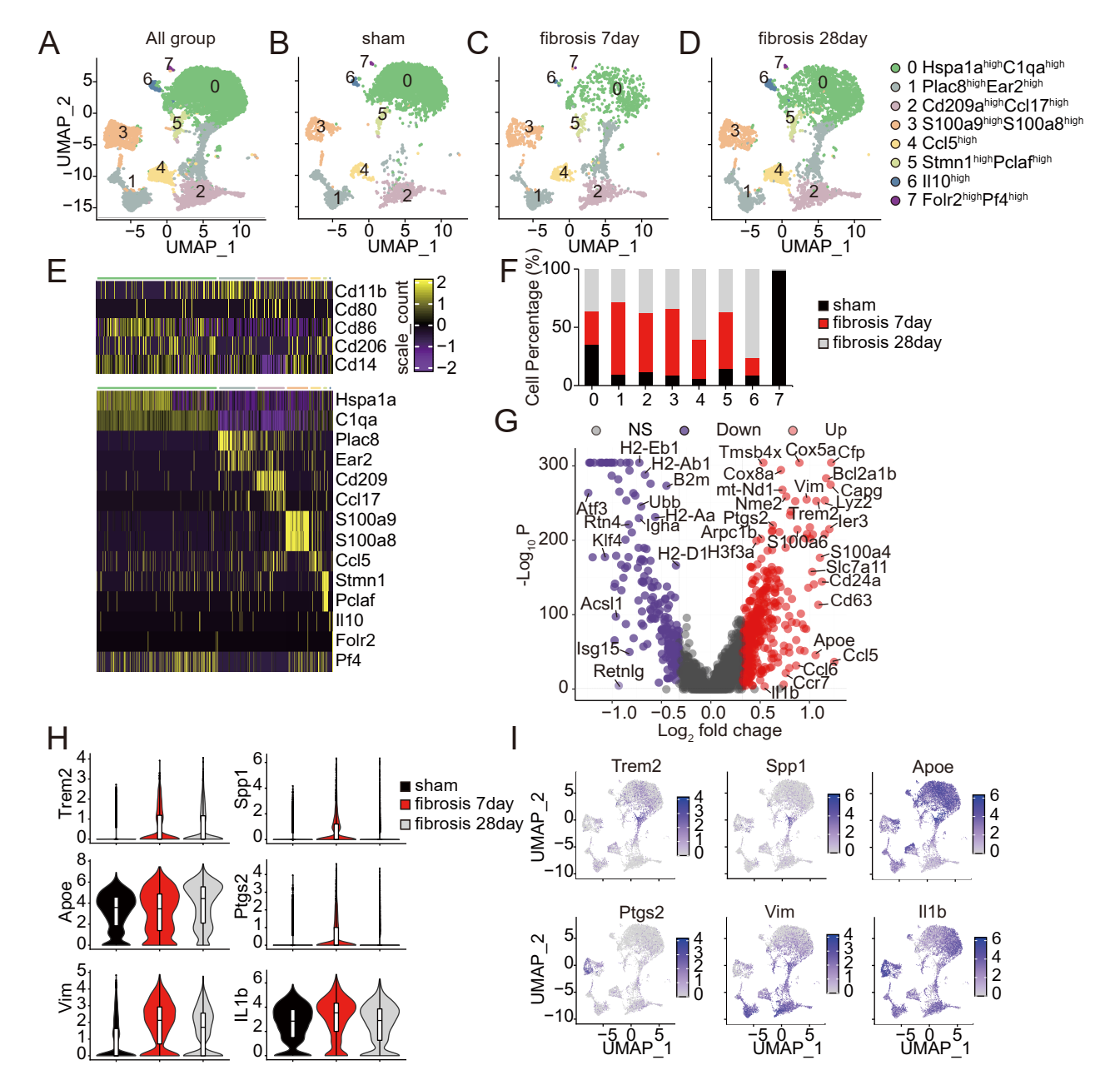
**Supplementary figure S1. scRNA-seq analysis discovered that Trem2 was significantly upregulated in macrophages from IRI-induced AKI-CKD transition mice.**

(A) scRNA-seq analysis of 12,014 macrophages from kidneys of sham, 7 days post IRI and 28 days post IRI mice. UMAP plot showing the macrophage clusters (0-7 clusters) in all groups (n=19).

(B) UMAP plot of macrophage clusters from sham mice (n=11).

(C) UMAP plot of macrophage clusters from 7 days post IRI mice (n=4).

(D) UMAP plot of macrophage clusters from 28 days post IRI mice (n=4).

(E) Heatmap of the identified markers of each macrophage clusters after de-batch analysis.

(F) Histogram of cell percentages in all macrophage clusters in three groups.

(G) Volcano plot of the differentially expressed genes (DEGs) in macrophages from two fibrosis mice compared to that of sham mice. The log_2_ fold change cut-off was 0.32, and the adjusted P value was 0.05.

(H) Violin plot of the expression levels of DEGs in three groups.

(I) Feature plot of the cluster-specific expression pattern of DEGs in three groups.

**
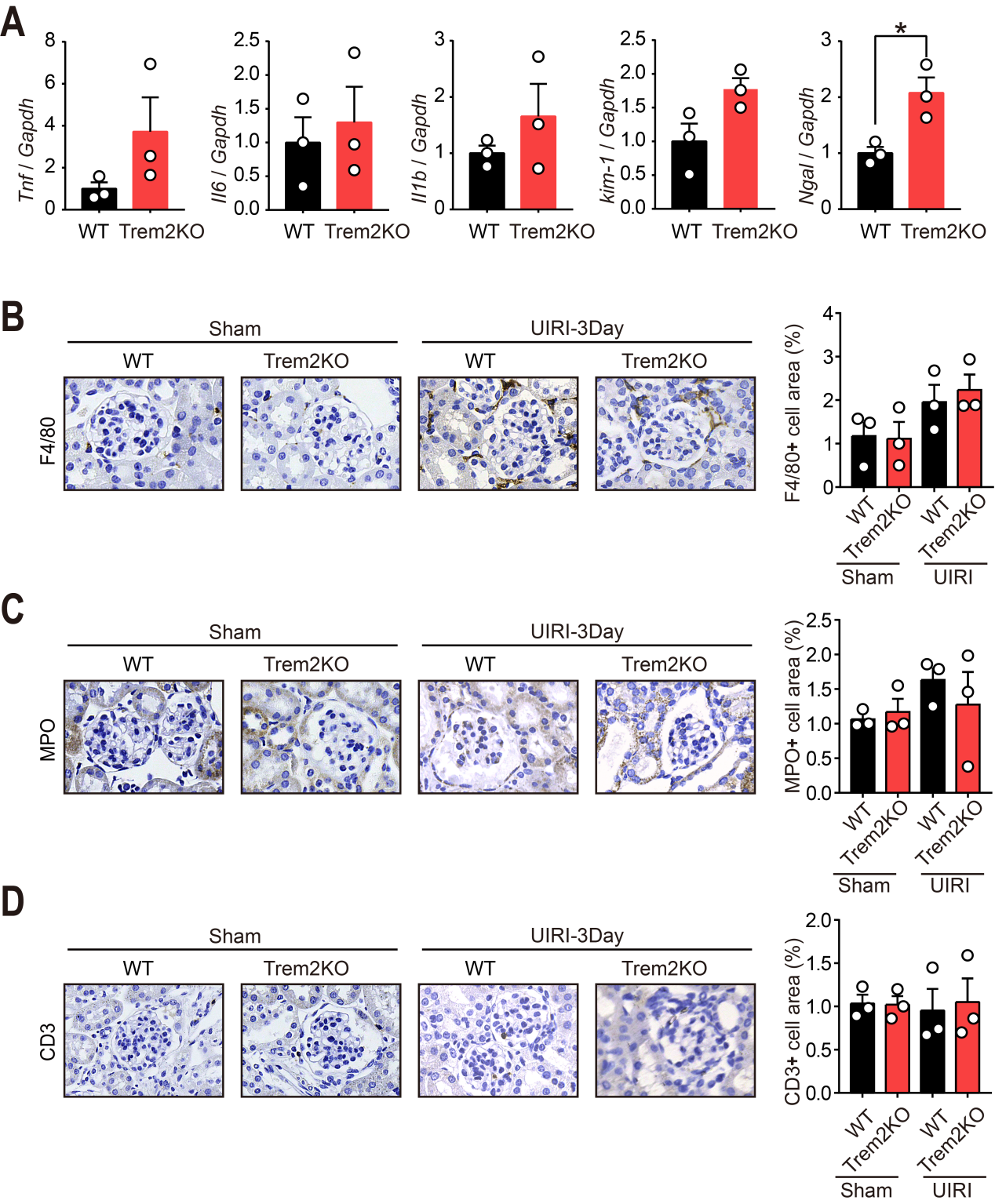
Supplementary figure S2.**

(A) qPCR results of *Tnf*, *Il6*, *Il1b*, *Kim-1*, and *Ngal* mRNA levels in kidneys from WT and Trem2KO mice on day 3 post-UIRI. n >= 4 per group. Mean ± SEM. *p < 0.05.

(B-D) IHC staining images and quantification results of F4/80+ macrophages (B), MPO+ neutrophils (C), and CD3+ T cells (D) in the glomerular regions of kidneys from WT and Trem2KO mice on day 3 post-sham and -UIRI. n = 3 per group. Scale bar 50 μm. Mean ± SEM. *p < 0.05.

**Supplementary figure S3.**

(A) Flow cytometry analysis of the F4/80+CD11b+ macrophage numbers in kidneys from four groups on day 14. n = 3 per group. Mean ± SEM.

(B) Flow cytometry of ROS levels in WT and Trem2KO BMDMs after HR treatment. Mean ± SEM. *p < 0.05.

(C) Flow cytometry of phagocytic levels in WT and Trem2KO BMDMs after HR treatment. Mean ± SEM. *p < 0.05.

(D) Flow cytometry of TREM2+ BMDMs after HR treatment in the presence of DMSO (Con) and HIF1a inhibitor LW6. n = 3 per group. Mean ± SEM. ***p < 0.001.

**
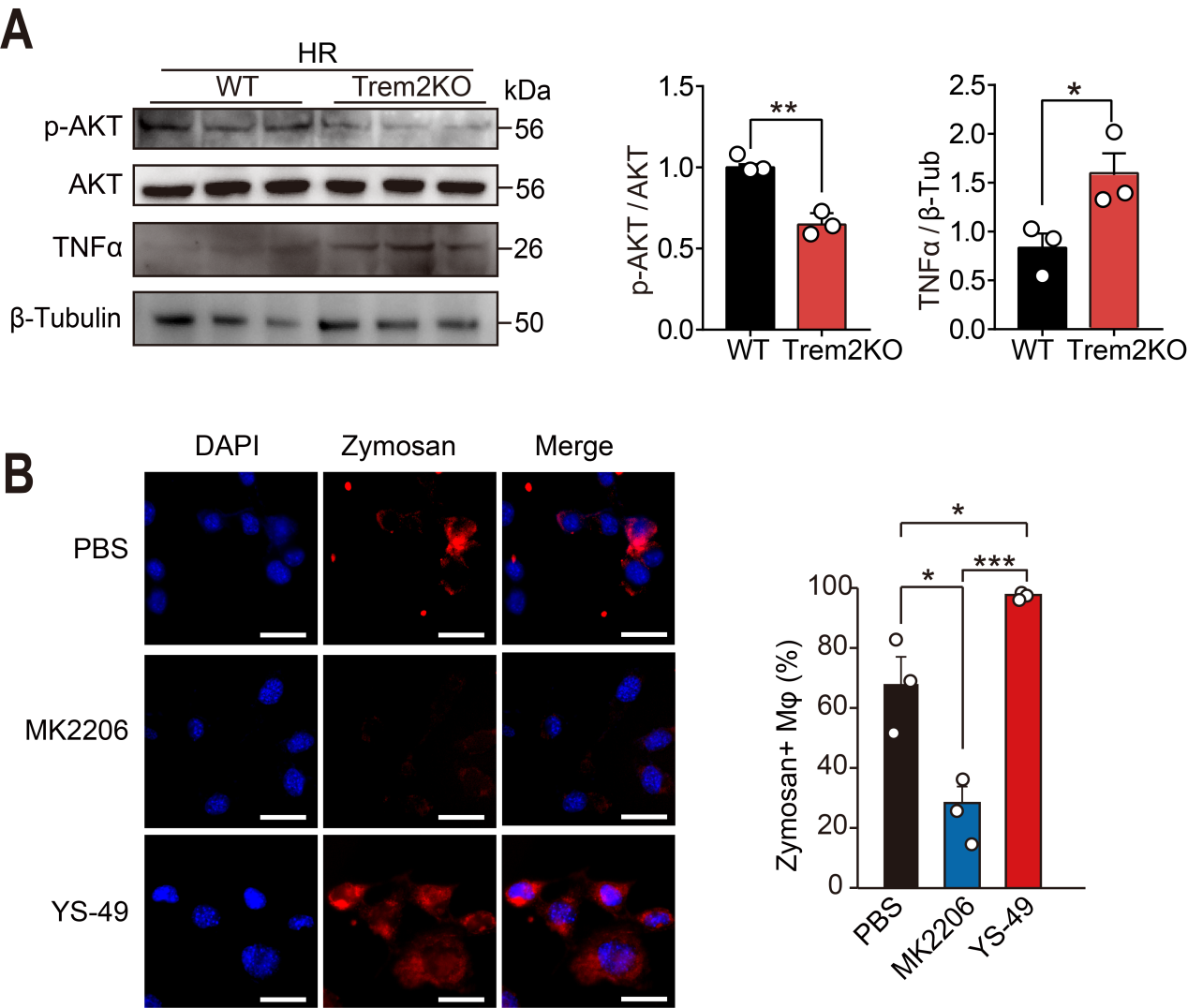
**

**Supplementary figure S4.**

(A) Western blotting results of p-AKT, AKT, and TNFα protein levels in WT BMDMs treated with HR in the presence and absence of a p-AKT inhibitor MK2206 for 24 hours. n = 3 per group. Mean ± SEM. *p < 0.05.

(B) Phagocytic levels of BMDMs treated with PBS, MK2206, and YS-49. n = 3 per group. Mean ±SEM. *p < 0.05, ***p < 0.001.

**
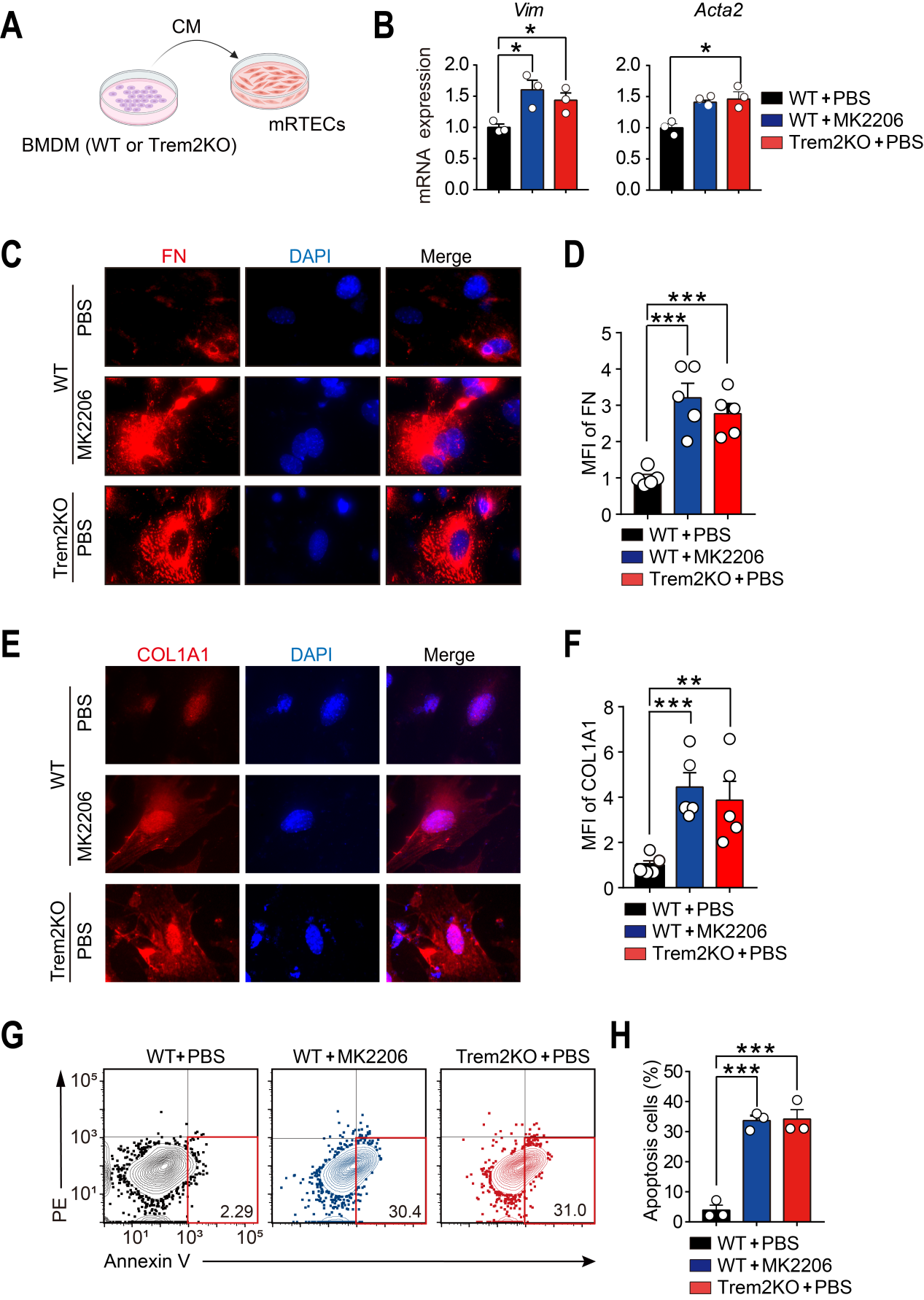
**

**Supplementary figure S5. Trem2KO BMDMs increased the renal tubular epithelial cell injury *in vitro*.**

(A) Schematic of the experimental design. After HR stimulation, conditioned medium (CM) from WT and Trem2KO BMDMs was added to the mouse primary renal tubular epithelial cells (mRTECs) and incbuated for 48 hours.

(B) qPCR results of the *Vim* and *Fn* mRNA levels in mRTECs after the incubation with the CM from HR-treated WT and Trem2KO BMDMs. n = 3 per group. Mean ± SEM. *p < 0.05.

(C and D) Immunostaining images and quantification result of the fibronectin (FN) expression in mRTECs after the incubation with the CM from HR + PBS-treated WT, HR + MK2206-treated WT, and HR + PBS-treated Trem2KO BMDMs. n = 5 per group. Mean ± SEM. ***p < 0.001.

(E and F) Immunostaining images and quantification result of the collagen type 1 (Col1) expression in mRTECs after the incubation with the CM from three groups of BMDMs. n = 5 per group. Mean ± SEM. **p < 0.01, ***p < 0.001.

(G and H) Flow cytometry analysis of the apoptosis in mRTECs after the incubation with the CM from three groups. n = 3 per group. Mean ± SEM. ***p < 0.001.

| **Gene** | **Species** | **Forward (5'-3')** | **Reverse (5'-3')** |
| --- | --- | --- | --- |
| *Gapdh* | mouse | TGACCTCAACTACATGGTCTACA | CTTCCCATTCTCGGCCTTG |
| *Tnfα* | mouse | CAGGCGGTGCCTATGTCTC | CGATCACCCCGAAGTTCAGTAG |
| *Il1β* | mouse | GAAATGCCACCTTTTGACAGTG | TGGATGCTCTCATCAGGACAG |
| *Vimentin* | mouse | TCCACACGCACCTACAGTCT | CCGAGGACCGGGTCACATA |
| *Il6* | mouse | CTGCAAGAGACTTCCATCCAG | AGTGGTATAGACAGGTCTGTTGG |
| *Il10* | mouse | CTTACTGACTGGCATGAGGATCA | GCAGCTCTAGGAGCATGTGG |
| *Acta2* | mouse | CTGACAGAGGCACCACTGAA | AGAGGCATAGAGGGACAGCA |
| *Col1* | mouse | TGAACGTGGTGTACAAGGTC | CCATCTTTACCAGGAGAACCAT |
| *Trem2* | mouse | TGGGTCACCTCTAGCCTACC | AGGATGCTGGCTGCAAGAAA |

**Supplementary Table 1. Primer information.**

| **Reagent** | **Catalog number** | **Company** |
| --- | --- | --- |
| Anti-mouse IgG（HRP） | 7076S | CST |
| Anti-rabbit IgG,（HRP） | 7074 | CST |
| Anti-PI3K | 4228 | CST |
| Anti-TNF | ab1793 | Abcam |
| Anti-AKT | 4257 | CST |
| Anti-CD11b | 101229 | Biolegend |
| Anti-Vimentin | ab92547 | Abcam |
| Anti-Collagen I | ab260043 | Abcam |
| APC Rat Anti-CD11b | 553312 | BD |
| Anti-Fibronectin | 15613-1-AP | Proteintech |
| PE Rat Anti-Mouse F4/80 | 565410 | Abcam |
| Anti-GAPDH | 2118S | CST |
| h/mTREM2 APC MAb | FAB17291A | R&D |
| Anti-IL-1 beta | Ab283818 | Abcam |
| Anti-IL-6 | Ab290735 | Abcam |
| Mouse M-CSF Protein | 315-02 | PeproTech |
| Anti-F4/80 | ab300421 | Abcam |
| HRP-conjugated Beta Tubulin | HRP-66240 | Proteintech |
| Ms CD86 BV421 | 564198 | BD |

**Supplementary Table 2. Antibody information.**
