# Supplementary material for: An adoptive cell therapy with TREM2‐overexpressing macrophages mitigates the transition from acute kidney injury to chronic kidney disease
Source: Clin Transl Med. 2025 Feb 25;15(3):e70252. doi: 10.1002/ctm2.70252 (PMC11859120; doi:10.1002/ctm2.70252)
Supplement: Supplementary file 2 — Supporting Information [file CTM2-15-e70252-s001.docx]

| *Gapdh* | mouse | TGACCTCAACTACATGGTCTACA | CTTCCCATTCTCGGCCTTG |
| --- | --- | --- | --- |
| *Tnfα* | mouse | CAGGCGGTGCCTATGTCTC | CGATCACCCCGAAGTTCAGTAG |
| *Il1β* | mouse | GAAATGCCACCTTTTGACAGTG | TGGATGCTCTCATCAGGACAG |
| *Vimentin* | mouse | TCCACACGCACCTACAGTCT | CCGAGGACCGGGTCACATA |
| *Il6* | mouse | CTGCAAGAGACTTCCATCCAG | AGTGGTATAGACAGGTCTGTTGG |
| *Il10* | mouse | CTTACTGACTGGCATGAGGATCA | GCAGCTCTAGGAGCATGTGG |
| *Acta2* | mouse | CTGACAGAGGCACCACTGAA | AGAGGCATAGAGGGACAGCA |
| *Col1* | mouse | TGAACGTGGTGTACAAGGTC | CCATCTTTACCAGGAGAACCAT |
| *Trem2* | mouse | TGGGTCACCTCTAGCCTACC | AGGATGCTGGCTGCAAGAAA |
| *Ngal* | mouse | ATGTCACCTCCATCCTGGTCAG | GCCACTTGCACATTGTAGCTCTG AG |
| *Havcr1* | mouse | CTGGAATGGCACTGTGACATCCG | GCAGATGCCAACATAGAAGCCCCCCCCCCCCC |

**Supplementary Table 1. Primer information.**

| **Reagent** | **Catalog number** | **Company** |
| --- | --- | --- |
| Anti-mouse IgG（HRP） | 7076S | CST |
| Anti-rabbit IgG,（HRP） | 7074 | CST |
| Anti-PI3K | 4228 | CST |
| Anti-TNF | ab1793 | Abcam |
| Anti-AKT | 4257 | CST |
| Anti-CD11b | 101229 | Biolegend |
| Anti-Vimentin | ab92547 | Abcam |
| Anti-Collagen I | ab260043 | Abcam |
| APC Rat Anti-CD11b | 553312 | BD |
| Anti-Fibronectin | 15613-1-AP | Proteintech |
| PE Rat Anti-Mouse F4/80 | 565410 | Abcam |
| Anti-GAPDH | 2118S | CST |
| h/mTREM2 APC MAb | FAB17291A | R&D |
| Anti-IL-1 beta | Ab283818 | Abcam |
| Anti-IL-6 | Ab290735 | Abcam |
| Mouse M-CSF Protein | 315-02 | PeproTech |
| Anti-F4/80 | ab300421 | Abcam |
| HRP-conjugated Beta Tubulin | HRP-66240 | Proteintech |
| Ms CD86 BV421 | 564198 | BD |
| Anti-CD3 | GB13014-50 | Servicebio |
| Anti-Myeloperoxidase | GB150006-100 | Servicebio |

**Supplementary Table 2. Antibody information.**
